# Characterization of swallowing in older adults with dementia

**DOI:** 10.1590/2317-1782/e20230358en

**Published:** 2025-04-07

**Authors:** Bruna de Sousa Santos, Juliana Onofre de Lira, Laura Davison Mangilli

**Affiliations:** 1 Programa de Pós-graduação em Ciências da Reabilitação, Faculdade de Ciências e Tecnologias em Saúde, Universidade de Brasília – UnB, Brasília (DF), Brasil.

**Keywords:** Dementia, Dysphagia, Swallowing Disorders, Aging, Speech Therapy

## Abstract

**Purpose:**

To analyze swallowing in older adults with dementia through clinical evaluation at a referral center for elderly healthcare.

**Methods:**

Retrospective, cross-sectional, observational study with older people, stratified by the Clinical Dementia Rating (CDR). Characterization was based on data extracted from medical records, including functional, cognitive, and mood assessments. The clinical evaluation of swallowing consisted of food offered in three consistencies, analyzing 13 items and functional classification.

**Results:**

The sample included 149 older adults – 47 neurotypical (CDR 0), 37 with mild dementia (CDR 1), 40 with moderate dementia (CDR 2), and 25 with severe dementia (CDR 3). The groups differed significantly, indicating greater changes in swallowing according to the severity of dementia. For instance, CDR 3 had greater changes in oral spillage of liquids than CDR 0 (p=0.012*). Cough with solids and drop in oxygen saturation with liquids were greater in CDR 3 than in CDR 1 (p=0.039* and p=0.047*, respectively). CDR 3 also had a higher frequency of reduced laryngeal excursion with nectar than CDR 2 (p=0.044*). Only positive cervical auscultation with nectar showed greater change in CDR 2 than in CDR 1 (p=0.019*). Oral residue of solids had a greater change in CDR 1 than in CDR 0 (p=0.030*).

**Conclusion:**

The severity of dementia was associated with swallowing impairments, highlighting the need for specific interventions in this population.

## INTRODUCTION

Dementia is a degenerative neurological syndrome, currently recognized as one of the most common geriatric morbidities. It is characterized by cognitive decline and/or behavioral changes that impact the person’s functioning, excluding other neurological and psychiatric pathologies^(1)^. This condition manifests in different ways, with the most prevalent types being dementia due to Alzheimer's disease (AD) and vascular dementia (VD). The resulting impacts vary between mild, moderate, and severe, depending on the dementia progression stages^(2)^.

According to recent research, dementia ranks seventh among the leading causes of death, with approximately 57 million individuals living with the condition worldwide. Projections indicate that this number may triple by 2050, reaching 153 million cases^(3)^.

Eating and swallowing challenges commonly occur throughout the dementia process, varying according to the stage and specific type of dementia^(4)^. Cognitive decline due to dementia may create the need for partial or total assistance to eat^(5)^.

Dysphagia is a clinical condition involving difficulties in swallowing, affecting the safe passage of food and liquids from the mouth to the stomach. This condition can arise due to various causes, including neuromuscular disorders, structural lesions in the gastrointestinal tract, neurological diseases, and other etiologies^(6)^. A recent study found that older adults with dementia are prone to developing oropharyngeal dysphagia (OD) over the disease progression^(4)^. Some swallowing manifestations include decreased pharyngeal sensitivity, hyposalivation, tongue hypotonia, increased oral and pharyngeal transit time, reduced upper esophageal sphincter closure, delayed swallowing trigger, food refusal, and food neglect^(5,7)^.

As highlighted in another study^(8)^, dysphagia in older people with dementia is associated with complications, including dehydration, malnutrition, and recurrent respiratory infections, with a potentially fatal impact. Furthermore, the occurrence of dysphagia in this population is related to an unfavorable prognosis, contributing to longer hospital stays and increased health costs^(4)^. Investigations conducted by Marin et al.^(5)^ indicate that the caregivers’ difficulties in managing feeding can increase the risk of choking, prolong the time food remains in the mouth, and increase the likelihood of aspiration.

Newman et al.^(7)^ reviewed the literature and concluded that the severity of dysphagia increases as dementia progresses. They highlighted the need to adapt alternative feeding routes to reduce the risk of bronchoaspiration and malnutrition. Takizawa et al.^(9)^ conducted a systematic review to improve understanding and awareness of the prevalence of dysphagia in patients with dementia. They found discrepancies between studies, reflecting research gaps. They reported that the accurate diagnosis of dysphagia in older adults with dementia is a challenge because symptoms can be mistakenly attributed to other aspects of the disease, making it difficult to identify swallowing problems specifically. Moreover, the lack of standardized protocols for assessing swallowing in older people with dementia can lead to different research methods, making it difficult to compare and generalize the results.

Despite the information available in the literature, studies with more participants and a specific approach to swallowing assessment are still needed, considering the different types of dementia. It is essential to improve the approach to dysphagia, incorporating an assessment with a reliable and standardized method to identify swallowing changes and prevent underdiagnosis and its potential implications. Thus, this study analyzed in detail the characteristics of swallowing in each dementia stage.

Hence, this study aimed to analyze the swallowing of older people with dementia, through items of their clinical evaluation process at a university hospital’s referral center for elderly healthcare (CRASI, in Portuguese).

## METHODS

This retrospective, cross-sectional, observational study was conducted with secondary data. It was approved by the Research Ethics Committee of the School of Health Sciences and Technologies at the University of Brasília, under CAAE: 03055118.80000.8093 and evaluation report number 3.121.872.

The sample consisted of older adults with dementia and neurotypical ones. This convenience sample included all patients undergoing speech-language-hearing (SLH) screening at a CRASI in the Brazilian Federal District between September 2017 and December 2019 and who met the inclusion criteria. Most older adults were referred to CRASI from primary healthcare through the regulatory system to determine the clinical diagnosis or adapt the therapeutic approach. There were no restrictions on these patients’ admission to the service; however, due to the sector’s organization, most patients still had to have their dementia or other cognitive deficits clarified.

The study used the following inclusion criteria: age 60 years or older; having undergone the Clinical Dementia Rating (CDR) scale^(10)^; and having undergone multidisciplinary screening, including SLH screening. The exclusion criteria were as follows: missing data in the evaluation form, compromising the study interpretation and analysis; medical records that for some reason could not be retrieved from the archives; non-Brazilian individuals; individuals with very mild cognitive impairment (CDR score = 0.5); individuals who underwent head and neck surgery; and individuals who had previously undergone SLH treatment.

Participants were stratified into groups according to the CDR classification of dementia severity. The neurotypical group (CDR 0) consisted of older adults without neurological or psychiatric disorders, with a CDR score of 0. Individuals with dementia had a medical diagnosis according to criteria of the National Institute of Neurological and Communicative Diseases and Stroke – Alzheimer’s Disease and Related Disorders Association (NINCDS-ARDRA)^(2)^ and a CDR score of 1 (mild dementia) (CDR 1), 2 (moderate dementia) (CDR 2), or 3 (severe dementia) (CDR 3). Chart 1 presents the main cognitive and functional manifestations of each stage of dementia.

**Chart 1:** Characterization of dementia stages considering their cognitive and functional manifestations, according to the Clinical Dementia Rating (CDR)^(2)^

Function	**Level of functional impairment**				
	None (CDR 0)	Questionable (CDR 0.5)	Mild (CDR 1)	Moderate (CDR 2)	Severe (CDR 3)
Memory	No memory loss or slight inconsistent forgetfulness.	Mild consistent forgetfulness; partial recollection of events; "benign" forgetfulness.	Moderate memory loss; more marked for recent events; interferes with everyday activities.	Severe memory loss; only highly learned material retained; new material rapidly lost.	Severe memory loss; only fragments remain.
Orientation	Fully oriented.	Fully oriented except for slight difficulty with time relationships.	Moderate difficulty with time relationships; oriented for place and person at examination but may have geographic disorientation.	Severe difficulty with time relationships; usually disoriented to time, often to place.	Oriented to person only.
Judgment and problem solving	Solves everyday problems well; judgment good in relation to past performance.	Slight impairment in solving problems, similarities, and differences.	Moderate difficulty in handling complex problems; social judgment usually maintained.	Severely impaired in handling problems, similarities, and differences; social judgment usually impaired.	Unable to make judgments or solve problems.
Community affairs	Independent function at usual level in job, shopping, business, and financial affairs, volunteer and social groups.	Slight impairment in these activities.	Unable to function independently at these activities although may still be engaged in some; appears normal to casual inspection.	No pretense of independent function outside home; appears well enough to be taken to functions outside a family home.	No pretense of independent function outside home; appears too ill to be taken to functions outside a family home
Home and hobbies	Life at home, hobbies, and intellectual interests well maintained.	Life at home, hobbies, and intellectual interests slightly impaired.	Mild but definite impairment of function at home; more difficult chores abandoned; more complicated hobbies and interests abandoned.	Only simple chores preserved; very restricted interests, poorly maintained.	No significant function in home.
Personal care	Fully capable of self-care.		Needs prompting.	Requires assistance in dressing, hygiene, keeping of personal effects.	Requires much help with personal care; frequent incontinence.

Source: adapted from Macedo Montaño et al.^(10)^

The reception at the service lasted 30 to 50 minutes, carried out by a multidisciplinary team (nursing, physiotherapy, SLH, geriatrics, dentistry, and social services), in which each professional category conducted an initial approach, raising the specific issues of their area. This reception took place 7 or 15 days after the first consultation with the geriatrician. After going through all the specialties at the service, the older person was released with the necessary guidance, and the professionals went to the team meeting to discuss the cases, prepare the unique therapeutic projects, and refer patients to other services, when necessary.

This study collected data from the patients' physical records – comprehensive geriatric assessment (with identification data self-reported by the patient and/or caregiver and functional and cognitive assessments) and SLH screening (with data from the clinical evaluation of swallowing). The identification data included sociodemographic information (e.g., sex, age, education in years, and performance on tests assessing functional, cognitive, and emotional status). These tests include the Mini-Mental State Examination (MMSE), Clinical Dementia Rating (CDR), reduced Geriatric Depression Scale (GDS) for responsive older patients, Cornell Scale, Pfeffer Index, Katz Scale, Semantic Verbal Fluency (SVF), and Clock Drawing Test (CDT), as recommended by the Brazilian Academy of Neurology^(1)^ (Appendix 1).

This study examined the association between clinical, functional, and mood assessment performance and dementia severity, measured by the CDR. No correlation was performed between these instruments and the specific clinical swallowing assessment items, which description is in Appendix 2.

Data on the clinical evaluation of swallowing were taken from the SLH screening protocol (Appendix 3), an adapted version of the Clinical Evaluation of Swallowing, available in the I Brazilian Consensus on Nutrition and Dysphagia in Hospitalized Older People^(11)^ and the SLH Dysphagia Risk Evaluation Protocol (PARD, in Portuguese)^(12)^. During the evaluation, 200 ml of water at room temperature were offered, served in standard disposable cups, to assess the liquid consistency. For the nectar consistency, 100 ml of thickened liquid was made available according to the instructions on the product label, using the Nestlé Resource^®^ ThickenUp Clear thickener, adding 1.2 g of the thickener for every 100 ml of water at room temperature. The solid food was one cornstarch cracker. They were offered the nectar, solid, and liquid consistencies (in this order, according to the study’s protocol) to verify^(12)^ oral residue, abnormal utensil grasp, abnormal oral transit time, abnormal chewing, abnormal laryngeal excursion, nasal reflux, choking, throat clearing, coughing, wet voice, positive cervical auscultation, and drop in oxygen saturation (drop above 4%). These were classified following guidelines in the specialized literature, aligned with the PARD^(12)^, whose two-fold approach per item classifies them as absent or present, according to the clinical signs of dysphagia.

**Figure 1:**
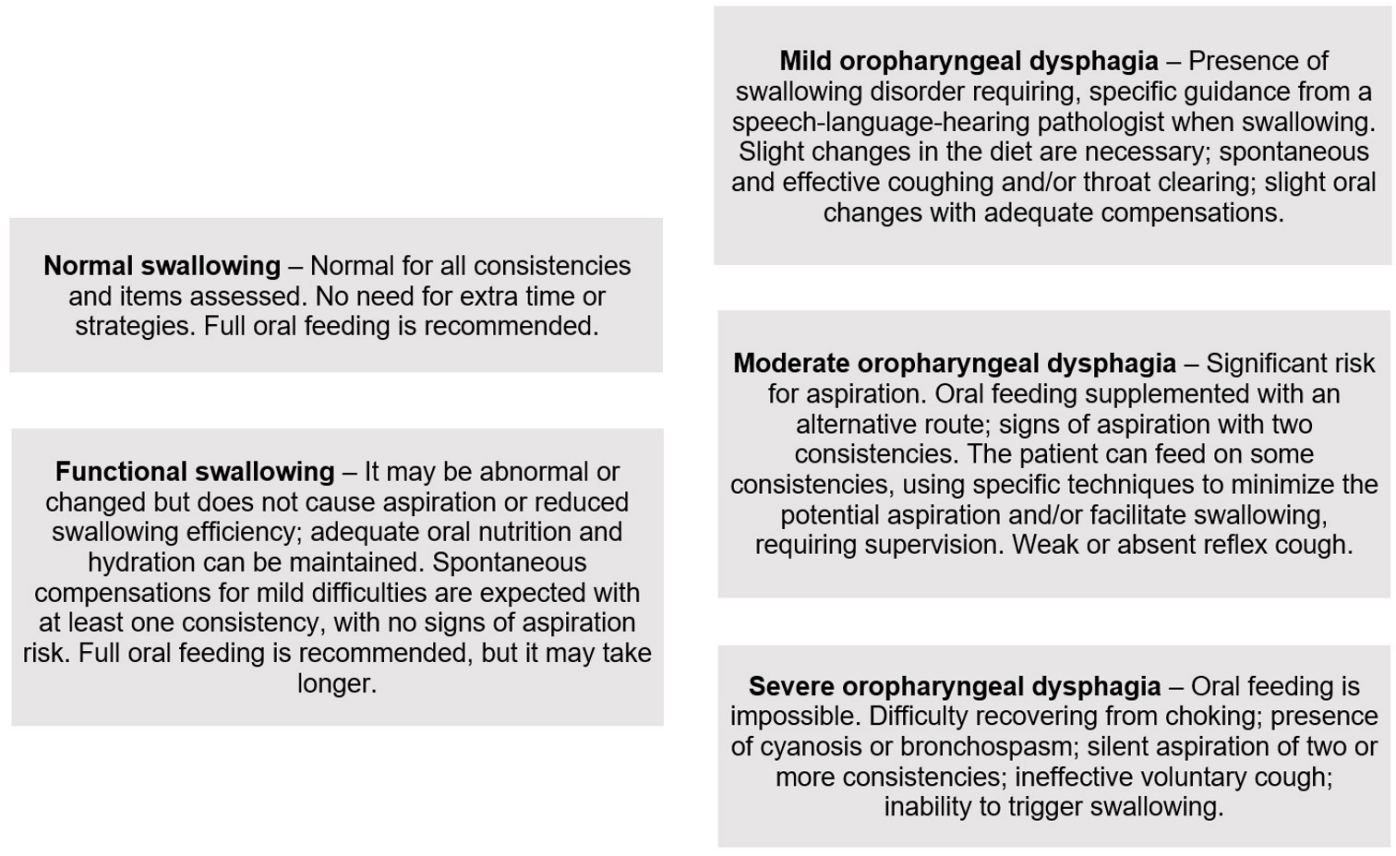
Functional classification of swallowing^(12)^

Source: Adapted from Padovani et al.^(12)^

Clinical evaluation of swallowing was a common practice at the SLH service, in which properly trained interns conducted the procedure under the supervision of the SLH pathologists in charge. Before starting the evaluation, they monitored the patients' vital signs, assessing oxygen saturation, heart rate, and respiratory rate. They also performed cervical auscultation to identify potential laryngeal noises indicative of laryngotracheal penetration or aspiration. An oximeter was used throughout the evaluation to detect possible episodes of bronchospasm associated with laryngotracheal aspiration, evidenced by the decrease in oxygen perfusion. As a standard service procedure, the cases were submitted for discussion after the clinical evaluation to define the clinical diagnosis of swallowing. The dysphagia classification in this study was based on the PARD^(12)^, which provides a standardized structure to evaluate and categorize swallowing disorders. The PARD uses the Dysphagia Outcome and Severity Scale and the Dysphagia Severity Scale to define seven distinct levels, each reflecting a specific degree of impairment and guiding/recommending therapeutic approaches. This study adapted the protocol to ensure clear and precise categorization – it did not use the intermediate classifications (mild to moderate; moderate to severe), whose presence may be associated with confounding factors^(13)^. The detailed criteria for dysphagia classification are presented in Figure 1.

### Data analysis

The data were analyzed descriptively and inferentially using SPSS 25.0 software. The significance level was set at 5% for inferential analyses. The descriptive analysis calculated measures of central tendency (mean) and variability (standard deviation) of quantitative variables and absolute and relative frequencies of qualitative variables.

Quantitative variables underwent normality analysis with the Kolmogorov-Smirnov test. Differences between data means were tested with the Student's t-test for independent samples (t) and its non-parametric counterpart, the Mann-Whitney test. The study presented parametric results when the two tests had similar results and non-parametric ones when they diverged.

The sample characterization was analyzed using all CDR groups with the chi-square test (X^2^) (without Yates correction) or Fisher's exact test (if the contingency table had any expected value lower than 5) for categorical variables. The study used the Analysis of Variance Test (ANOVA) for continuous variables, comparing pairs of means with the Tukey test (post hoc).

The dependent variables were the items of the clinical evaluation of swallowing per food consistency and the functional classification of swallowing. Their association with the severity of dementia (CDR) underwent inferential analysis with the chi-square test or Fisher's exact test, when necessary, with binary variables to capture the difference in expected values between CDR categories.

## RESULTS

The initial study sample had 230 individuals, but 81 were excluded for different reasons – 52 for having a CDR = 0.5, one for being under 60 years old, and 28 because their medical records could not be retrieved.

As shown in Table 1, the sample comprised 102 participants with dementia (68.4%) and 47 neurotypicals (31.5%). Females predominated in all groups. Their ages ranged from 60 to 92 years, with a mean of 74 years in neurotypical older people and 77 in those with dementia. Also, neurotypical older adults attended school for more years, on average, than those with dementia.

**Table 1:** Characterization of the sample according to sociodemographic variables, functional, cognitive, and mood assessment, and type of dementia in older adults, according to the CDR and its associations

		**CDR 0**	**CDR 1**	**CDR 2**	**CDR 3**	**Total**	**p**
**Total number of individuals (n/%)**		**47 (31.3%)**	**37 (24.6%)**	**40 (26.6%)**	**25 (17.3%)**		
Age in years (Mean ± SD)		74.49 ± 6.92	75.70 ± 8.42	77.03 ± 7.83	79.80 ± 5.74	-	0.033*
Education in years (Mean ± SD)		7.38 ± 6.05	4.43 ± 4.55	7.05 ± 5.35	6.60 ± 5.29	-	0.074
Females (n/%)		36 (76.6%)	29 (78.4%)	22 (55.0%)	18 (72%)	105 (70.9%)	0.086
Type of dementia (n/%)							<0.001*
Alzheimer’s disease		-	5 (13.5%)	10 (25.0%)	12 (48%)	27 (26.4%)	
Vascular dementia		-	10 (27%)	9 (22.5%)	11 (44%)	30 (29.4%)	
Mixed		-	2 (5.4%)	7 (17.5%)	0 (0%)	9 (8.82%)	
Under investigation		-	19 (51.4%)	14 (35.0%)	2 (8%)	35 (34.3%)	
Other*		-	1 (2.7%)	0 (0%)	0 (0%)	1 (0.98%)	
**Cognitive, functional, and mood assessment instruments**							
MMSE	Abnormal	24 (51.1%)	35 (94.6%)	40 (100%)	23 (100%)	132 (89.7%)	<0.001*
	Total	47	37	40	23	147	
SVF (animals)	Abnormal	14 (31.8%)	21 (56.8%)	28 (75.7%)	18 (100%)	81 (60.4%)	<0.001*
	Total	44	35	37	18	134	
CDT	Abnormal	16 (38.1%)	18 (62.1%)	33 (82.5%)	14 (100%)	81 (66.9%)	<0.001*
	Total	42	29	36	14	121	
Pfeffer	Abnormal	7 (15.2%)	34 (97.1%)	37 (97.4%)	19 (95%)	97 (69.7%)	<0.001*
	Total	46	35	38	20	139	
Katz	D	3 (6.4%)	5 (13.9%)	12 (31.6%)	17 (73.9%)	37 (25.6%)	<0.001*
	PD	3 (6.4%)	5 (13.9%)	18 (47.4%)	5 (21.7%)	31 (21.5%)	
	I	41 (87.2%)	26 (72.2%)	8 (21.1%)	1 (4.3%)	76 (52.7%)	
	Total	47	36	38	23	144	
Depression	Yes	20 (43.5%)	22 (64.7%)	24 (64.9%)	7 (58.3%)	73 (56.5%)	0.159
	Total	46	34	37	12	129	

* Statistic significance (p<0,005)

**Caption:** SD = standard deviation; n = absolute frequency; % = relative frequency; MMSE = Mini-Mental State Examination; SVF = Semantic Verbal Fluency Test; CDT = Clock Drawing Test; D = dependent; PD = partially dependent; I = independent

Analysis of dementia classification indicated that most individuals in the CDR 1 group had not yet received a conclusive diagnosis (51.4%), with the type of dementia under investigation, followed by VD (27%), AD (13.5%), mixed dementia (5.4%), and one case of primary progressive aphasia (2.7%), a variant of frontotemporal dementia. Similarly, cases without a conclusive diagnosis predominated (35%) in the CDR 2 group, followed by AD (25%), VD (22.5%), and mixed dementia (17.5%). On the other hand, approximately half of the individuals in the CDR 3 group were diagnosed with AD (48%), followed by VD (44%), while only two lacked a complete diagnosis (8%).

Table 1 also shows that functional and cognitive assessment analysis found that changes began to manifest from the mild phase of dementia. The rates of change progressively increased as dementia progressed. In the CDR 2 group, the change in the MMSE reached 100%, indicating a generalized cognitive impairment. However, neurotypical individuals likewise had a significant prevalence of change. Moreover, both the SVF and the CDT showed a prevalence of change already in the mild phase of dementia, with change rates reaching 100% in the CDR 3 group, evidencing progressive cognitive deterioration throughout the evolution of dementia.

Table 2 presents the results of the clinical evaluation of swallowing in the four groups and shows which clinical signs of swallowing changes were statistically significantly different.

**Table 2:** Result of the descriptive analysis of the variables of the clinical evaluation of swallowing in relation to the CDR, according to food consistencies

**Item/consistency assessed**			**CDR**				
			**0**	**1**	**2**	**3**	**Overall frequency (n/%)**
Anterior oral spillage	Nectar	Present	2 (4.5%)	2 (5.6%)	1 (2.6%)	4 (17.4%)	9 (6.3%)
		Total	44	36	38	23	
	Solid	Present	2 (4.4%)	2 (5.7%)	4 (10%)	1 (5%)	9 (6.4%)
		Total	45	35	40	20	
	Liquid **^c,e^**	Present	3 (7.1%)	5 (14.3%)	3 (7.9%)	7 (31.8%)	18 (13.1%)
		Total	42	35	38	22	
Oral residue	Nectar **^c,e^**	Present	0 (0%)	1 (2.8%)	0 (0%)	4 (18.2%)	4 (2.8%)
		Total	44	36	37	23	
	Solid **^a^**	Present	6 (13.6%)	12 (34.3%)	11 (28.9%)	7 (38.9%)	36 (26.6%)
		Total	44	35	38	18	
	Liquid **^c,e,f^**	Present	0 (0%)	0 (0%)	0 (0%)	4 (18.2%)	4 (3%)
		Total	42	35	39	22	
Abnormal utensil grasp	Nectar	Present	3 (7%)	8 (22.2%)	9 (24.3%)	3 (13%)	23 (16.5%)
		Total	43	36	37	23	
	Solid	Present	7 (15.9%)	7 (20%)	11 (28.2%)	4 (20%)	29 (21%)
		Total	44	35	39	20	
	Liquid	Present	4 (9.5%)	7 (20%)	7 (18.4%)	6 (27.3%)	24 (17.5%)
		Total	42	35	38	22	
Abnormal oral transit time	Nectar	Present	3 (6.8%)	8 (22.9%)	8 (21.1%)	4 (18.2%)	23 (16.5%)
		Total	44	35	38	22	
	Solid	Present	4 (8.9%)	8 (22.9%)	9 (23.7%)	5 (26.3%)	26 (18.9%)
		Total	45	35	38	19	
	Liquid **^c^**	Present	4 (9.8%)	6 (17.1%)	6 (16.2%)	9 (40.9%)	25 (18.5%)
		Total	41	35	37	22	
Abnormal chewing	Solid	Present	13 (29.5%)	16 (45.7%)	19 (48.7%)	8 (40%)	44 (31.8%)
		Total	44	35	39	20	
Reduced laryngeal excursion	Nectar **^b^**	Present	4 (9.3%)	7 (19.4%)	10(27%)	3(13.6%)	24 (17.3%)
		Total	43	36	37	22	
	Solid	Present	5 (11.1%)	6 (17.1%)	9 (23.1%)	5 (25%)	25 (17.9%)
		Total	45	35	39	20	
	Liquid	Present	6 (14.6%)	6 (17.1%)	8 (21.8%)	6 (26.1%)	26 (18.7%)
		Total	41	35	38	23	
Nasal reflux	Nectar	Present	2 (4.5%)	0 (0%)	0 (0%)	1 (4.5%)	3 (2.1%)
		Total	44	36	37	22	
	Solid	Present	2 (4.4%)	0 (0%)	0 (0%)	0 (0%)	2 (1.4%)
		Total	45	35	40	20	
	Liquid	Present	2 (4.8%)	1 (2.9%)	0 (0%)	1 (4.3%)	4 (2.8%)
		Total	42	35	39	23	
Choking	Nectar	Present	2 (4.4%)	0 (0%)	1 (2.7%)	2 (8.7%)	5 (3.5%)
		Total	44	36	37	23	
	Solid	Present	2 (4.4%)	0 (0%)	0 (0%)	1 (5%)	3 (2.1%)
		Total	45	35	40	20	
	Liquid	Present	3 (7.1%)	1 (2.9%)	3 (7.7%)	4 (17.4%)	11 (7.9%)
		Total	42	35	39	23	
Throat clearing	Nectar	Present	4 (9.1%)	4 (11.1%)	7 (18.9%)	4 (17.4%)	19 (13.5%)
		Total	44	36	37	23	
	Solid	Present	4 (8.9%)	4 (11.4%)	9 (22.5%)	2 (10%)	19 (13.5%)
		Total	45	35	40	20	
	Liquid **^b^**	Present	2 (4.9%)	4 (11.4%)	8 (21.1%)	4 (17.4%)	18 (13.1%)
		Total	41	35	38	23	
Cough	Nectar	Present	2 (4.5%)	0 (0%)	2 (5.4%)	1 (4.3%)	5 (3.5%)
		Total	44	36	37	23	
	Solid **^f^**	Present	2 (4.4%)	0 (0%)	3 (7.9%)	3 (15%)	8 (5.7%)
		Total	45	35	38	20	
	Liquid ^b^	Present	2 (4.8%)	2 (5.7%)	8 (21.1%)	4 (17.4%)	16 (11.5%)
		Total	42	35	38	23	
Wet voice	Nectar	Present	10 (23.3%)	13 (36.1%)	6 (16.2%)	5 (23.8%)	34 (24.8%)
		Total	43	36	37	21	
	Solid	Present	9 (20%)	7 (20%)	7 (17.9%)	2 (10.5%)	25 (18.1%)
		Total	45	35	39	19	
	Liquid	Present	9 (21.4%)	13 (37.1%)	11 (28.9%)	6 (31.6%)	39 (29.1%)
		Total	42	35	38	19	
Positive cervical auscultation	Nectar **^b,c,d^**	Present	2 (4.5%)	3 (8.3%)	12 (32.4%)	5 (23.8%)	25 (18.1%)
		Total	44	36	37	21	
	Solid	Present	5 (11.1%)	6 (17.1%)	6 (15.4%)	5 (27.8%)	22 (15.9%)
		Total	45	35	39	18	
	Liquid **^b,c^**	Present	2 (4.8%)	5 (14.3%)	12 (31.6%)	7 (31.8%)	26 (18.9%)
		Total	42	35	38	22	
Drop in oxygen saturation	Nectar	Present	3 (7.7%)	1 (3.3%)	1 (2.9%)	1 (5.9%)	7 (5.8%)
		Total	39	30	34	17	
	Solid	Present	2 (5.1%)	1 (3.3%)	1 (3%)	2 (13.3%)	6 (5.1%)
		Total	39	30	33	15	
	Liquid **^c,f^**	Present	0 (0%)	0 (0%)	2 (6.1%)	3 (15.8%)	5 (4.1%)
		Total	38	30	33	19	

Pearson chi-square test

**Caption:** n = absolute frequency; % = relative frequency; a = statistically significant result between CDR 0 and CDR 1; b = statistically significant result between CDR 0 and CDR 2; c = statistically significant result between CDR 0 and CDR 3; d = statistically significant result between CDR 1 and CDR 2; e = statistically significant result between CDR 2 and CDR 3; f = statistically significant result between CDR 1 and CDR 3

Most variables with a statistically significant difference showed a greater change in CDR 3 in relation to CDR 0 – oral spillage of liquid (p = 0.012), oral residue of nectar (p = 0.010), oral residue of liquid (p = 0.010), abnormal oral transit time of liquid (p = 0.015), positive cervical auscultation with nectar (p = 0.026), positive cervical auscultation with liquid (p = 0.013), and drop in oxygen saturation with liquid (p = 0.029). CDR 3 had greater changes than CDR 1 in the oral residue of liquid (p = 0.016), cough with solid (p = 0.039), and drop in oxygen saturation with liquid (p = 0.047).

Only oral residue of solids had greater changes in the CDR 1 group than in CDR 0 (p = 0.030).

CDR 0 was statistically significantly different from CDR 2, with greater changes in CDR 2 in the reduced laryngeal excursion with nectar (p = 0.044), throat clearing with liquid (p = 0.043), coughing with liquid (p = 0.041), positive cervical auscultation with nectar (p = 0.001), and cervical auscultation with liquid (p = 0.002).

CDR 1 was statistically significantly different from CDR 2 in positive cervical auscultation with nectar, with greater changes in CDR 2 (p = 0.019).

CDR 2 was statistically significantly different from CDR 3, with greater changes in CDR 3 in oral spillage of liquid (p = 0.026), oral residue of nectar (p = 0.016), and oral residue of liquid (p = 0.012).

CDR 1 was statistically different from CDR 3, with greater changes in CDR 3 in oral spillage of liquid (p = 0.016), cough with solid (p = 0.039), and drop in oxygen saturation with liquid (p = 0.047).

As shown in Table 3, the functional swallowing classification demonstrated a statistical difference between normal swallowing, moderate dysphagia, and severe dysphagia between the groups. Functional swallowing (p = 0.520) and mild dysphagia (0.103) were statistically equivalent between the groups. However, the group-by-group analysis found that CDR 0 was statistically different from CDR 2 regarding mild dysphagia.

**Table 3:** Result of descriptive analysis of the functional classification of swallowing with dementia staging

	**CDR**				
	**0 (N = 41)**	**1 (N = 31)**	**2 (N = 35)**	**3 (N = 23)**	**Total**
	**n (%)**	**n (%)**	**n (%)**	**n (%)**	**n (%)**
Normal swallowing **^a,b,c^**	30 (73.2%)	13 (41.9%)	13 (37.1%)	6 (26.1%)	62 (48%)
Functional swallowing	9 (22.0%)	3 (9.7%)	5 (14.3%)	3 (13.0%)	20 (15.5%)
Mild dysphagia **^b^**	1 (2.4%)	4 (12.9%)	7 (20.0%)	4 (17.4%)	16 (12.4%)
Moderate dysphagia **^a,b^**	0 (0%)	8 (25.8%)	10 (28.6%)	2 (8.7%)	20 (15.5%)
Severe dysphagia **^c,e,f^**	1 (2.4%)	2 (6.5%)	0 (0%)	8 (34.8%)	11 (8.5%)

Pearson chi-square test analysis

**Caption:** N = number of participants per group according to CDR; n = absolute frequency; % = relative frequency; a = statistically significant result between CDR 0 and CDR 1; b = statistically significant result between CDR 0 and CDR 2; c = statistically significant result between CDR 0 and CDR 3; d = statistically significant result between CDR 1 and CDR 2; e = statistically significant result between CDR 2 and CDR 3; f = statistically significant result between CDR 1 and CDR 3

Furthermore, the group comparison found CDR 0 was statistically significantly different from the other groups, in that CDR 0 was associated with having normal swallowing, and the other groups were not – CDR 1 (p = 0.007), CDR 2 (p = 0.002), and CDR 3 (p < 0.001).

CDR 1 was associated with the presence of mild dysphagia when compared to CDR 0 (p = 0.021). CDR 2 was associated with the presence of mild dysphagia, and CDR 0 with its absence (p = 0.021). CDR 2 was also associated with the presence of moderate dysphagia, and CDR 0 with its absence (p < 0.001).

CDR 3 was associated with the presence of severe dysphagia when compared to CDR 0 (p < 0.001), CDR 1 (p = 0.011), and CDR 2 (p < 0.001).

## DISCUSSION

The main finding of this study was the presence of dysphagia in all stages of dementia. Studies address swallowing in older adults with dementia heterogeneously. Some focused on characterizing eating challenges in individuals with Alzheimer's disease^(14)^, while others sought to assess the risk of dysphagia associated with Alzheimer's disease^(15)^. Some studies addressed the prevalence of dysphagia in dementia contexts^(16)^, and others used objective measures to assess swallowing^(17-19)^.

Studies with patients with Alzheimer's disease are more prevalent, as it is the most common type of dementia. Few studies have investigated swallowing covering all stages of dementia and presented the results stratified per disease stage^(8,16,20)^. Considering the need for a more specific approach to swallowing in the mild, moderate, and severe stages of dementia, this study aimed to characterize these changes according to the dementia stages. It also established a group of neurotypical older people for comparison with those with dementia, following some previous studies’ approaches^(14,15,21)^.

### Sociodemographic characteristics and type of dementia

This study found a predominance of females, attributable to the feminization of aging and women’s greater life expectancy, as they live approximately 8 years longer than men^(22)^. Although no statistically significant difference was identified by sex in the different stages of dementia, the literature reports a higher prevalence of dementia in women in all stages of the disease^(8,14,16,18-20)^.

Also, individuals with dementia were older than neurotypicals. Statistical correlations established that patients’ ages increased as dementia progressed. This result was expected since dementia predominates in older adults, and the oldest ones are more likely to develop dementia^(22)^. Additionally, the mean age of participants with dementia was higher than that of neurotypical participants, agreeing with results from similar studies^(14,21)^.

No differences in education were observed in relation to the dementia stage. However, the literature highlights that low education is a predictive factor for the development of dementia since adequate levels of education help increase synaptic density and compensation for possible intellectual deficits^(2)^. It is important to note that the swallowing assessment used in this study is not linked to the participants’ education level, as they did not have to understand complex verbal or written commands for feeding.

VD was the most common dementia type in the sample, representing 29.4%, followed by AD, with 26.4%, mixed dementia, with 8.82%, and other types of dementia, with 0.98%. This study used the category "others" due to the low incidence of this type in the sample, with only one individual having primary progressive aphasia. These results contrast with epidemiological and clinical studies that indicate a higher prevalence of AD, followed by VD^(2,3)^.

Studies exploring the relationship between dementia and swallowing also present discrepant results compared to the findings of this study. A study with older adults with dementia^(19)^ reported that 54.4% of the sample had AD, with only 20.6% diagnosed with VD. Furthermore, 22.9% of the participants had Parkinson's dementia, a condition not found in the present study. On the other hand, another study identified a higher frequency of AD (52.9%) and mixed dementia (25.1%), with only 3.1% of VD^(8)^.

It is essential to highlight that most participants in our study diagnosed with dementia were still undergoing medical investigation to determine the specific type.

### Functional, cognitive, and mood aspects

The CDR assesses individuals comprehensively. Therefore, the other cognitive screening and functional and mood assessment instruments were used only to characterize the study sample. No correlations were made between performance on these tools and the clinical swallowing assessment items.

Most study participants were patients with dementia, with a predominance of the moderate phase (26.6%), followed by the mild (24.6%) and severe phases (17.3%). Although the distribution of dementia phases in the literature is heterogeneous, our findings are similar to those of previous studies^(16,18)^.

The MMSE cutoff scores were adjusted in this study according to the education level. These adjustments were also applied to the other cognitive and functional instruments in this sample, considering the sociocultural and educational diversity of the Brazilian population^(1,22)^. The adaptation based on education level aimed to avoid false-positive and false-negative evaluation results.

This study found that performance in functional and cognitive assessments was statistically significantly correlated with dementia severity, corroborating findings in the literature^(2)^. The groups with dementia had a prevalence of changes in the MMSE, SVF (animals), CDT, and Pfeffer, indicating that the sample mostly comprised older adults with lower functioning and greater dependence.

### Clinical evaluation of swallowing

This study detected swallowing changes with an adapted clinical evaluation protocol, observing several items during the ingestion of different food consistencies.

Clinical evaluation of swallowing involves the observation of clinical signs indicative of dysphagia and is frequently used, especially in hospital settings. It is worth mentioning that this is a noninvasive, easy-to-reproduce, low-cost approach^(23)^. Despite recognizing these merits, discussions on the effectiveness of clinical evaluation in the literature are limited, considering instrumental methods (e.g., videofluoroscopy and videoendoscopy of swallowing) as the most reliable for identifying changes in swallowing^(24)^. However, instrumental examinations in specific cases, such as dementia, can be challenging since they require adequate attention levels, preserved oral comprehension, and the patient’s cooperation^(5)^.

This study found that older adults with mild dementia had a higher frequency of solid residue in the oral cavity than neurotypical ones. This finding can be explained by the decreased tongue strength, reduced sensitivity in the oral cavity and pharynx, and impaired oral motor coordination – characteristics associated with dementia, as reported in the literature^(25)^. No disparities were identified in this specific clinical evaluation component in the other stages of dementia. A plausible explanation for this pattern is that individuals who have been facing the disease for longer commonly have dietary restrictions of solid foods, being frequently associated with choking, swallowing difficulties, and oral health deterioration^(26)^.

The results of this study are similar to the findings of another one with 26 older women with mild and moderate AD, which identified oral residue of pureed and solid foods (34.6%)^(17)^. However, direct comparison between studies is limited due to the different stratification of stages, types of dementia, and approaches to consistencies.

The study observed reduced laryngeal excursion during swallowing of nectar, throat clearing and coughing with liquids, and positive cervical auscultation with nectar and liquids in the moderate phase of dementia compared to neurotypical individuals. The change identified in our research may be associated with the greater viscosity of nectar compared to liquid, requiring greater force to swallow^(27)^.

The observation of greater force required to swallow nectar compared to thinner liquids highlights a relevant aspect in dysphagia associated with dementia, particularly in the moderate phase. Because nectar is thicker, it requires a more intense muscular response during swallowing. This additional demand for force may be attributed to the need to overcome its viscosity, making it more challenging to move the bolus^(27)^.

Significant changes occur throughout the aging process in structures crucial for swallowing, including decreased tone of intrinsic and extrinsic laryngeal muscles and ossification of laryngeal cartilages, reducing laryngeal excursion^(4,6)^. These changes are exacerbated in cases of associated dementia^(16,17)^. It is important to emphasize that the laryngeal excursion mechanism plays a fundamental role in swallowing, acting in coordination with the hyoid bone to ensure airway protection, contributing to safe and efficient swallowing. Thus, changes in this mechanism may be associated with bronchoaspiration^(6)^.

In this study, coughing when ingesting liquids was associated with moderate dementia. The presence of coughing during or after swallowing suggests possible aspiration or penetration of food and/or liquids, triggering laryngeal sensitivity, and stimulating the protective reflex of the lower airways to expel the content^(6)^. Coughing occurs often in older people, and when associated with dementia, it highlights the difficulty in swallowing due to cognitive decline and deficits in oral motor function^(8)^.

The study observed a series of swallowing changes in the severe phase of dementia, including prevalence of anterior oral spillage of liquid, oral residue of nectar and liquid, increased oral transit time of liquids, cough with solids, positive cervical auscultation with nectar and liquid, and a drop in oxygen saturation with liquid.

The significant presence of anterior oral spillage was specifically associated with severe dementia, although it was also identified in other dementia stages. A previous study that used videofluoroscopy in subjects with different types of dementia in the moderate and severe stages reported changes related to oral spillage in 24% of the sample^(19)^.

Another study explained the association between anterior oral spillage and severe dementia by apathy in more advanced dementia stages. Apathy can negatively influence the speed of the anticipatory and oral preparatory phases of swallowing, which, in turn, facilitates food spillage from the mouth^(5)^.

Abnormal oral transit time was associated with severe dementia, indicating a proportional relationship between such time and the CDR evolution. A previous study with older individuals diagnosed with AD corroborates these findings, showing a significant increase in oral transit time in severe dementia^(20)^. This prolonged time can be attributed to orotactile agnosia, executive dysfunctions, and decreased tongue strength. Orotactile agnosia, manifested by reduced sensory input in the oral cavity, hinders food perception during the preparatory and oral phases of swallowing^(25)^.

Coughing in moderate and severe stages of dementia indicates the onset of laryngotracheal penetration and/or aspiration with these food consistencies^(15)^. Some studies indicate adapting textures and changing consistencies, especially thickening thin liquids, to promote safe and efficient swallowing in patients with oropharyngeal dysphagia and dementia^(8)^. These findings possibly explain the lack of statistical difference in the occurrence of coughing with nectar in this study.

The detection of positive cervical auscultation with nectar and liquid in individuals with severe dementia reveals relevant clinical implications, indicating a possible link between advanced cognitive impairment and swallowing dysfunctions^(15)^. This suggests the presence of laryngotracheal aspiration, increasing the risk of respiratory complications, such as pneumonia. Challenges in the anticipatory and preparatory phases of swallowing in severe dementia are notable, resulting in ineffective coordination between the orofacial muscles and the larynx^(7)^. Early identification of positive cervical auscultation highlights the importance of clinical evaluation of swallowing in this population.

Although there was no statistical significance in this study, grasping utensils properly during meals is a fine motor skill that can be affected by dementia, impairing autonomy and quality of life^(5)^. Individuals with dementia may have slow movements and lack hand dexterity. As dementia progresses, memory loss and reduced planning ability can make them forget how to use utensils correctly, making it difficult to organize the movements necessary for eating^(26)^.

Chewing should also be highlighted, although it was likewise not statistically different between the groups. Difficulties in chewing solids are common in early dementia stages, influenced by cognitive deterioration that compromises coordination between the jaw muscles and the tongue^(26)^. As dementia progresses, additional challenges arise, such as problems in identifying and properly handling food, resulting in chewing difficulties and an increased risk of aspiration^(25,26)^. Adapting the room where they have meals and modifying food consistency (e.g., serving softer options) are essential strategies to facilitate chewing in individuals with dementia^(8)^.

Nasal reflux was not associated with dementia in this study. This relationship has not been widely explored, and there is currently no substantial evidence to establish a direct link between nasal reflux and dementia.

### Functional classification of swallowing

The functional classification of swallowing shows that older adults with no neurological impairments had normal swallowing (as shown in Table 3). On the other hand, those with mild dementia had moderate dysphagia while participants with moderate dementia had varying degrees of dysphagia, ranging from mild to moderate. Lastly, older people with severe dementia had severe dysphagia.

The literature focused on swallowing in dementia highlights deteriorated swallowing functioning and an increased risk of dysphagia as dementia progresses^(9)^. The authors of a study with clinical evaluation found a 16.7% prevalence of dysphagia in individuals with mild and moderate AD, increasing to 91.8% in the severe phase^(15)^. Other authors^(21)^ investigated electrophysiological parameters of swallowing in all AD phases, concluding that AD patients had electrophysiological characteristics indicative of dysphagia even in the early stages of the disease.

Comparison of this study with others in the literature is limited due to disparities in the sample and swallowing assessment instruments. The results and associations are inferential, deriving from similarities and statistical analyses.

The present study has some limitations. First, the lack of previous studies using the same instrument to assess swallowing in older adults with dementia limits our ability to compare, as previous studies used different assessment methods. Furthermore, this study could not analyze swallowing changes separately per dementia type. It would be challenging to generalize the results to each specific category, given the diversity of dementia types in our sample.

The lack of a specific and validated instrument for the clinical assessment of swallowing in older adults with dementia is another relevant limitation. Moreover, a substantial part of our sample was being investigated, without a definitive diagnosis of dementia. This compromises the accurate characterization of the sample, limiting the generalization of the results to older people with dementia in general.

This set of limitations emphasizes the continuous need for further research to overcome methodological challenges and contributes to a more comprehensive and accurate understanding of swallowing disorders in older adults with dementia.

## CONCLUSION

This study identified distinct patterns of swallowing functioning at different dementia stages, assessed with the CDR. The highlights in each CDR were as follows:

- CDR 0 (neurotypical): normal swallowing;
- CDR 1 (mild dementia): Presence of oral residue of solid food. Associated with mild dysphagia;
- CDR 2 (moderate dementia): Reduced laryngeal excursion with nectar, throat clearing with liquids, coughing with liquids, and positive cervical auscultation with nectar. Associated with mild and moderate dysphagia;
- CDR 3 (severe dementia): Presence of anterior oral spillage of liquids, oral residue of nectar and liquids, abnormal oral transit time of liquids, abnormal cervical auscultation with nectar and liquids, coughing with solids, and a drop in oxygen saturation with liquids. Associated with severe dysphagia.

In summary, this study provides evidence of an association between dementia severity (as assessed with the CDR) and deteriorated swallowing function. These findings have crucial clinical implications for the management of patients with dementia, highlighting the importance of early assessment and intervention in dysphagia to improve quality of life and prevent complications associated with feeding and swallowing in this group.

## Appendix 1 Description of functional, cognitive, and mood assessment instruments, as recommended by the Brazilian Academy of Neurology^(1,2)^.

**Table:**

**Instrument**	**Description**
Mini-Mental State Examination	Cognitive assessment instrument to assess mental state and identify possible cognitive deficits. Its questions and tasks cover different cognitive aspects such as temporal and spatial orientation, memory, attention, and language. Its maximum score is 30 points. Higher scores indicate better cognitive functioning while lower scores may suggest cognitive impairment. For a more specific interpretation, this study established cutoffs based on the person's education level: 20 points for illiterates, 25 points for those with 1 to 4 years of education, 26 points for those with 5 to 8 years of education, 28 points for those with 9 to 11 years of education, and 29 points for those with over 11 years of education.
Clinical Dementia Rating (CDR)	A clinical tool used to assess the severity of dementia. This scale, used as a staging instrument, allows us to understand the progression and intensity of cognitive symptoms in individuals with dementia. By assigning scores in several domains, including memory, orientation, judgment, and communication, the CDR classifies patients as 0 (neurotypical), 0.5 (very mild cognitive impairment), 1 (mild dementia), 2 (moderate dementia), and 3 (severe dementia).
Geriatric Depression Scale (GDS)	An instrument used to assess depressive symptoms in older adults. Composed of a series of yes/no questions, the GDS aims to identify indicators of depression, such as discouragement, sadness, and lack of interest in daily activities. Higher scores reflect greater severity of depressive symptoms. The scale ranges from 0 to 15, and a score ≥ 5 suggests the possibility of depression.
Cornell Scale	An instrument designed to assess behavioral symptoms in older people with dementia. Composed of a series of questions about behaviors observed in the previous 7 days, the scale is answered by a caregiver or family member. The items cover areas such as aggression, resistance to care, agitation, depression, hallucinations, delusions, and so forth. The total score ranges from 0 to 45, with higher scores indicating greater severity of behavioral symptoms.
Pfeffer Index	Assessment tool that investigates older people’s ability to perform instrumental activities of daily living (IADLs). It consists of an interview applied to their caregiver or family member, addressing issues related to autonomy in daily tasks, such as financial management, shopping, telephone use, medication administration, and so on. Responses vary on a scale that includes the options "Yes, he/she is capable" (0); "Never did, but could do now" (0); "With some difficulty, but does" (1); "Never did and would have difficulty now" (1); "Needs help" (2); and "Not capable" (3). A total score equal to or greater than 6 points suggests dependence in IADLs.
Katz Scale	A tool used to assess functional independence in basic activities of daily living (BADL). Composed of six items that cover tasks such as bathing, dressing, going to the bathroom, transferring, continence, and eating, the scale classifies the person's performance in each category as independent, with some assistance, or dependent. Scores range from 0 to 6, indicating their level of functional independence, in which: (0) independent in all six functions; (1) independent in five functions and dependent in one; (2) independent in four functions and dependent in two; (3) independent in three functions and dependent in three; (4) independent in two functions and dependent in four; (5) independent in one function and dependent in five; (6) dependent in all six functions.
Semantic Verbal Fluency Test (SVF) – animals	A task used to assess cognitive function, especially language and semantic memory. The participant is instructed to name as many animals as possible in a specific time, usually 1 minute. Performance on the task reflects their ability to recall words associated with the topic and measures verbal fluency and semantic organization. The total number of animals named is counted to determine the score on the task. Cutoff scores are 9 points for illiterates, 12 points for 1 to 8 years of schooling, and 13 points for 9 or more years of schooling.
Clock Drawing Test (CDT)	Assessment instrument that covers memory, motor function, executive function, and verbal comprehension. The patient is instructed to draw a clock face, indicating the hands at 11:00 and 10:00. The version by Shulman et al, (1993) establishes a score of 0 to 5 points, as follows: (0) inability to represent the clock; (1) clock-related drawing, but with severe visuospatial disorganization; (2) moderate visuospatial disorganization with incorrect timekeeping, perseveration, left-right confusion, missing and/or repeated numbers, lack or excess of hands; (3) correct visuospatial distribution with incorrect timekeeping; (4) small spatial errors with correct digits and time; (5) perfect clock.

## Appendix 2 Clinical swallowing assessment items, adapted from Padovani et al.^(12)^

**Table:**

**Item assessed**	**Description**
Anterior oral spillage	It is considered present when food or liquid flows through the corners of the mouth after the bolus has been captured, generally due to insufficient lip sealing.
Abnormal utensil grasp	It is considered present when postural compensations or a lack of precision occurs when the individual takes a utensil (such as a cup, spoon, or fork) to the mouth to capture the food.
Oral residue	It is the accumulation of food in the anterior vestibule, lateral vestibule, floor of the mouth, and/or tongue surface after swallowing. It is defined as present when food residues are found in the oral cavity after swallowing, corresponding to more than approximately 25% of the volume offered.
Abnormal oral transit time	It is considered present when the time between the complete capture of the food bolus and the beginning of the elevation of the hyolaryngeal complex, determined by the triggering of the swallowing reflex, exceeds 4 seconds.
Wet voice	It is considered present when bubbling sounds are produced during prolonged phonation of the vowel "e", after offering a food consistency. This phenomenon indicates the stasis of secretions, liquids, or food in the laryngeal vestibule and may indicate silent penetration into the vocal folds.
Reduced laryngeal excursion	It is considered present when there is limited capacity for anterior and superior laryngeal elevation (visually less than two fingers of the examiner) during swallowing, which indicates an increased risk of aspiration.
Nasal reflux	It is considered present when the consistency returns to the nasal cavity during swallowing, resulting from insufficient velopharyngeal closure.
Choking	Defined as partial or complete airflow obstruction due to the entry of a foreign body into the lower airways. It is considered present when there is choking with difficult recovery, and coughing during swallowing; cyanosis may occur, with difficult recovery of the baseline respiratory rate.
Throat clearing	The sensation or action of the vocal folds coming together, making an "ahem" sound, usually due to the presence of mucus and/or food in the vocal fold region.
Cough	Reflex coughing during or after swallowing is a sign of aspiration, indicating laryngeal sensitivity and the ability to expectorate. It is considered present when there is an unprompted cough before, during, or after swallowing.
Positive cervical auscultation	It is considered present when there are noises in breathing before swallowing that remain with the same frequency after offering a food consistency. It is also considered present if there are noises after swallowing that were not present before.
Drop in oxygen saturation	It is considered present when there is a reduction of more than 4% in arterial oxygenation in relation to the basal value after offering a food consistency. This is based on the hypothesis that food aspiration can trigger bronchospasm reflex, reducing ventilation-perfusion efficiency, and resulting in decreased oxygen saturation.
Abnormal chewing	It is considered present when there is any change in the usual ability to grind food into smaller particles, facilitating swallowing and digestion. This change can manifest through uncoordinated jaw movements during chewing and asymmetry in jaw movements during the process.

## Appendix 3 Clinical swallowing assessment protocol used in the service

**Table:**

*CLINICAL SWALLOWING ASSESSMENT: [acronyms for filling in items: P-present A-absent]*			
- Pre-assessment laryngeal auscultation: ⬜ negative ⬜ positive ___________________________________			
- Pre-assessment vital signs HR: ___ bpm (60 to 100 bpm) RR: ___ rpm (12 to 20 rpm) SpO_2_: ____ % (> 90%)			
**Events present**	**Nectar**	**Solid**	**Liquid**
Anterior oral spillage			
Oral residue			
Abnormal utensil grasp			
Abnormal oral transit time			
Abnormal chewing			
Reduced laryngeal excursion			
Nasal reflux			
Choking			
Throat clearing			
Cough			
Wet voice			
Positive cervical auscultation			
SpO_2_ (drop greater than 4%)			
Recovery to base saturation: ( ) Quick ( ) Slow			
Post-assessment vital signs: HR: _______ RR: ________ SpO_2_: ___________			
*CONCLUSION – SPEECH-LANGUAGE-HEARING ASSESSMENT:*			
- Functional classification of swallowing -			
Normal ⬜ functional ⬜ (spontaneous compensations for mild difficulties with at least one consistency, with no signs of aspiration risk) Mild dysphagia ⬜ Moderate dysphagia ⬜ Severe dysphagia ⬜			

## References

[1] Smid J, Studart-Neto A, César-Freitas KG, Dourado MCN, Kochhann R, Barbosa BJAP (2022). Declínio cognitivo subjetivo, comprometimento cognitivo leve e demência - diagnóstico sindrômico: recomendações do Departamento Científico de Neurologia Cognitiva e do Envelhecimento da Academia Brasileira de Neurologia. Dement Neuropsychol.

[2] Schilling LP, Balthazar MLF, Radanovic M, Forlenza OV, Silage ML, Smid J (2022). Diagnóstico da doença de Alzheimer: recomendações do Departamento Científico de Neurologia Cognitiva e do Envelhecimento da Academia Brasileira de Neurologia. Dement Neuropsychol.

[3] Suemoto CK, Mukadam N, Brucki SMD, Caramelli P, Nitrini R, Laks J (2023). Risk factors for dementia in Brazil: differences by region and race. Alzheimers Dement.

[4] De Stefano A, Di Giovanni P, Kulamarva G, Gennachi S, Di Fonzo F, Sallustio V (2020). Oropharyngeal dysphagia in elderly population suffering from mild cognitive impairment and mild dementia: understanding the link. Am J Otolaryngol.

[5] Marin SMC, Mansur LL, Oliveira FF, Marin LF, Wajman JR, Bahia VS (2021). Swallowing in behavioral variant frontotemporal dementia. Arq Neuropsiquiatr.

[6] Campos SML, Trindade DRP, Cavalcanti RVA, Taveira KVM, Ferreira LMBM, Magalhães HV (2022). Sinais e sintomas de disfagia orofaríngea em idosos institucionalizados: revisão integrativa. Audiol Commun Res.

[7] Newman RD, Ray R, Woodward L, Glass B (2020). Factors contributing to the preferred method of feeding in end‑stage dementia: a scoping review. Dysphagia.

[8] Espinosa-Val MC, Martín-Martínez A, Graupera M, Arias O, Elvira A, Cabré M (2020). Prevalence, risk factors, and complications of oropharyngeal dysphagia in older patients with dementia. Nutrients.

[9] Takizawa C, Gemmell E, Kenworthy J, Speyer R (2016). A systematic review of the prevalence of oropharyngeal dysphagia in stroke, Parkinson’s Disease, Alzheimer’s Disease, head injury, and pneumonia. Dysphagia.

[10] Macedo Montaño MBM, Ramos LR (2005). Validade da versão em português da Clinical Dementia Rating. Rev Saude Publica.

[11] SBGG: Sociedade Brasileira de Geriatria e Gerontologia (2011). I Consenso Brasileiro de Nutrição e Disfagia em Idosos Hospitalizados..

[12] Padovani AR, Moraes DP, Mangili LD, de Andrade CRF (2007). Protocolo fonoaudiológico de avaliação do risco para disfagia (PARD). Rev Soc Bras Fonoaudiol.

[13] Payten CL, Chiapello G, Weir KA, Madill CJ (2024). Frameworks, terminology and definitions used for the classification of voice disorders: a scoping review. J Voice.

[14] Kai K, Hashimoto M, Amano K, Tanaka H, Fukuhara R, Ikeda M (2015). Relationship between eating disturbance and dementia severity in patients with Alzheimer’s disease. PLoS One.

[15] Simões ALS, Oliva A, Hebling E (2020). Signs for early detection of dysphagia in older adults with severe Alzheimer’s Disease. J Nutr Health Aging.

[16] Michel A, Vérin E, Gbaguidi X, Druesne L, Roca F, Chassagne P (2018). Oropharyngeal Dysphagia in Community-Dwelling Older Patients with Dementia: Prevalence and Relationship with Geriatric Parameters. J Am Med Dir Assoc.

[17] Sanches EP, Bilton T, Ramos LR (2000). Análise descritiva da alimentação de idosos com demência. Distúrb Comun.

[18] Özsürekci C, Arslan SS, Demir N, Çalışkan H, Şengül Ayçiçek G, Kılınç HE (2020). Timing of Dysphagia Screening in Alzheimer’s Dementia. JPEN J Parenter Enteral Nutr.

[19] Feinberg MJ, Ekberg O, Segall L, Tully J (1992). Deglutition in elderly patients with dementia: findings of videofluorographic evaluation and impact on staging and management. Radiology.

[20] Dias MC, Vicente LCC, Friche AAL, Ribeiro EG, Motta AR (2018). Tempo de trânsito oral na demência de Alzheimer. Audiol Commun Res.

[21] Seçil Y, Arıcı Ş, İncesu TK, Gürgör N, Beckmann Y, Ertekin C (2016). Dysphagia in Alzheimer’s disease. Neurophysiol Clin.

[22] Santos CS, Bessa TA, Xavier AJ (2020). Fatores associados à demência em idosos. Ciênc Saúde Coletiva.

[23] Balbinot J, Machado GC, Hübner LS, Real CS, Signorini AV, Dornelles S (2019). Protocolos de avaliação da deglutição: Norteadores e limitações. Clin Biomed Res.

[24] Dantas RO (2021). Videofluoroscopia na avaliação da deglutição (en la evaluación de la deglución). Salud(i)Ciencia.

[25] Ji EK, Wang HH, Jung SJ, Lee KB, Kim JS, Hong BY (2019). The changes for strength of oropharyngeal muscles in patients with dementia and dysphagia. Brain Neurorehabil.

[26] Moraes MS, Canuto MSB, Moreira LYA (2023). A dentição do idoso e as implicações alimentares. Distúrb Comun.

[27] Madalozzo B, Aoki MCS, Soria F, Santos RS, Furkim AM (2017). Análise acústica do tempo de deglutição através do Sonar Doppler. Rev CEFAC.

