# ERRATUM: Characterization of swallowing in older adults with dementia

**DOI:** 10.1590/2317-1782/e20230183en

**Published:** 2025-06-16

Due to an honest mistake by the authors, the article “Characterization of swallowing in older adults with dementia” (DOI https://doi.org/10.1590/2317-1782/e20230358en), present in CoDAS 2025;37(3):e20230358, was published without identifying all authors.

**Where it was previously identified:**

Bruna de Sousa Santos^1^https://orcid.org/0000-0002-6695-9615

Juliana Onofre de Lira^1^https://orcid.org/0000-0001-8938-4781

Laura Davison Mangilli^1^https://orcid.org/0000-0003-2739-126X

Now identify:

Bruna de Sousa Santos^1^https://orcid.org/0000-0002-6695-9615

Juliana Onofre de Lira^1^https://orcid.org/0000-0001-8938-4781

Cristina Lemos Barbosa Furia^1^https://orcid.org/0000-0001-9507-6072

Laura Davison Mangilli^1^https://orcid.org/0000-0003-2739-126X

**Where it was previously identified:**

**Author contributions**

Conception and design of the study: BSS, JOL and LDM; Data collection, analysis and interpretation: BSS, JOL and LDM; Writing or revising the article in an intellectually important way: BSS, JOL and LDM; Final approval of the version to be published: JOL and LDM.

**Now identify:**

**Author contributions**

Conception and design of the study: BSS, JOL, CLBF and LDM; Data collection, analysis and interpretation: BSS, JOL, CLBF and LDM; Writing or revising the article in an intellectually important way: BSS, JOL, CLBF and LDM; Final approval of the version to be published: JOL and LDM.

The authors apologize for the error.

