# Supplementary material for: Characterization of swallowing in older adults with dementia
Source: Codas. 2025 Apr 7;37(3):e20230358. doi: 10.1590/2317-1782/e20230358en (PMC11991718; doi:10.1590/2317-1782/e20230358en)
Supplement: Translation

# Translation: Caracterização da deglutição de idosos com demência

**DOI:** 10.1590/2317-1782/e20230358pt

**Authors:** Bruna de Sousa Santos, Juliana Onofre de Lira, Laura Davison Mangilli

**Affiliations:** 1 Programa de Pós-graduação em Ciências da Reabilitação, Faculdade de Ciências e Tecnologias em Saúde, Universidade de Brasília – UnB, Brasília (DF), Brasil.

**Keywords:** Demência, Disfagia, Transtornos de Deglutição, Envelhecimento, Fonoaudiologia

## Abstract

**Objetivo:**

Analisar a função de deglutição em idosos com demência através de avaliação clínica em um Centro de Referência em Assistência à Saúde do Idoso.

**Método:**

Estudo observacional transversal retrospectivo com idosos, estratificados através do Clinical Dementia Rating (CDR). A caracterização ocorreu a partir de dados extraídos dos prontuários e que incluíram avaliação funcional, cognitiva e de humor. A avaliação clínica da deglutição consistiu na oferta de alimentos em três consistências, analisando treze itens e classificação funcional.

**Resultados:**

Participaram 149 idosos: 47 neurotípicos (CDR 0), 37 com demência leve (CDR 1), 40 com demência moderada (CDR 2) e 25 com demência grave (CDR 3). Houve diferenças significativas entre os grupos, indicando maior alteração na deglutição conforme a gravidade da demência. Por exemplo, o CDR 3 apresentou maior alteração para escape oral para líquidos em comparação ao CDR 0 (p=0,012*). Tosse para sólidos e queda na saturação de oxigênio para líquidos foram maiores no CDR 3 do que CDR 1 (p=0,039* e p=0,047*, respectivamente). O CDR 3 também mostrou maior frequência de excursão laríngea reduzida para néctar em comparação ao CDR 2 (p=0,044*). Apenas a ausculta cervical positiva para néctar mostrou maior alteração no CDR 2 do que CDR 1 (p=0,019*). O resíduo oral para sólidos mostrou maior alteração no CDR 1 comparado ao CDR 0 (p=0,030*).

**Conclusão:**

Houve associação entre a gravidade da demência e comprometimentos na deglutição, evidenciando a necessidade de intervenções específicas nessa população.

## INTRODUÇÃO

A demência é uma síndrome neurológica degenerativa, atualmente reconhecida como uma das morbidades geriátricas mais comuns. Caracteriza-se por um declínio cognitivo e/ou alterações comportamentais que impactam na funcionalidade do indivíduo, excluindo outras patologias neurológicas e psiquiátricas^(1)^. Essa condição apresenta manifestações diversas, sendo os tipos mais prevalentes a demência por doença de Alzheimer (DA) e demência vascular (DV). Os impactos decorrentes manifestam-se de formas variadas, conforme os estágios de progressão da demência, podendo ser leve, moderado e grave^(2)^.

De acordo com pesquisas recentes, a demência ocupa a sétima posição entre as principais causas de mortalidade, com aproximadamente 57 milhões de indivíduos vivendo com essa condição globalmente. Projeções indicam que até 2050 esse número pode triplicar, atingindo a marca de 153 milhões de casos^(3)^.

Ao longo do processo demencial, é comum a manifestação de desafios relacionados à alimentação e deglutição, variando de acordo com o estágio e o tipo específico de demência^(4)^. O declínio cognitivo presente nos casos demenciais pode imputar necessidade de assistência parcial ou total para se alimentar^(5)^.

A disfagia é uma condição clínica que envolve dificuldades na deglutição, afetando a passagem segura dos alimentos e líquidos da boca até o estômago. Essa condição pode surgir devido a uma variedade de causas, incluindo distúrbios neuromusculares, lesões estruturais no trato gastrointestinal, doenças neurológicas e outras etiologias^(6)^. Em uma pesquisa recente, foi constatado que idosos com demência têm propensão a desenvolver disfagia orofaríngea (DO) ao longo da progressão da doença^(4)^. Algumas manifestações da deglutição incluem a diminuição da sensibilidade na região faríngea, hipossalivação, hipotonia da língua, aumento do tempo de trânsito oral e faríngeo, redução do fechamento do esfíncter esofágico superior, atraso no disparo da deglutição, recusa alimentar e negligência em relação ao alimento^(5,7)^.

Conforme destacado em outro estudo^(8)^, a presença de disfagia em idosos com demência está associada a complicações, incluindo desidratação, desnutrição e infecções respiratórias recorrentes, com potencial impacto fatal. Além disso, a ocorrência de disfagia nessa população está relacionada a um prognóstico desfavorável, contribuindo para um prolongamento no tempo de internação hospitalar e um aumento nos custos relacionados à saúde^(4)^. As investigações conduzidas por Marin e colaboradores^(5)^ indicam que as dificuldades enfrentadas pelos cuidadores no manejo da alimentação podem acentuar o risco de engasgo, prolongar o tempo de permanência do alimento na boca e elevar a probabilidade de aspiração.

Newman e colaboradores^(7)^ revisaram a literatura e concluíram que a gravidade da disfagia aumenta à medida que a demência progride. Destacaram a necessidade de adaptação de vias alternativas de alimentação para reduzir o risco de broncoaspiração e desnutrição. Takizawa e colaboradores^(9)^ realizaram uma revisão sistemática com o objetivo de melhorar a compreensão e a conscientização sobre a prevalência de disfagia em pacientes com demência. Concluiu-se que há discrepâncias entre os estudos, refletindo em lacunas na pesquisa. Relatam que o diagnóstico preciso da disfagia em idosos com demência é um desafio, já que os sintomas podem ser atribuídos erroneamente a outros aspectos da doença, dificultando a identificação específica dos problemas de deglutição. Além disso, a falta de protocolos padronizados para avaliação da deglutição em idosos com demência pode levar a uma variabilidade nos métodos de pesquisa, tornando difícil comparar e generalizar os resultados.

Apesar das informações disponíveis na literatura, ainda é necessário estudos que envolvam um maior número de participantes, que tenham uma abordagem específica da avaliação da deglutição, e que considerem os diversos tipos de demência. É fundamental aprimorar a abordagem da disfagia, incorporando uma avaliação por meio de método confiável e padronizado para identificar alterações da deglutição, prevenindo assim o subdiagnóstico e suas potenciais implicações. Desta forma, o presente estudo oferece uma análise detalhada das características da deglutição em cada fase da demência.

Diante do exposto, o objetivo deste estudo é analisar a função de deglutição em idosos com demência, por meio dos itens que compõem seu processo de avaliação clínica, assistidos em um Centro de Referência em Assistência à Saúde do Idoso (CRASI) de um hospital universitário.

## MÉTODO

Foi conduzido um estudo observacional transversal retrospectivo, realizado mediante consulta de dados secundários. Foi aprovado pelo Comitê de Ética e Pesquisa da Faculdade de Ciências e Tecnologias em Saúde da Universidade de Brasília, sob o CAAE: 03055118.80000.8093 e parecer de aprovação número 3.121.872.

A amostra foi composta por idosos neurotípicos e idosos com demência. Trata-se de uma amostragem por conveniência que compreendeu todos os pacientes que passaram pela triagem fonoaudiológica em um CRASI no Distrito Federal, no período entre setembro de 2017 e dezembro de 2019, e que atenderam aos critérios de inclusão. Os idosos foram encaminhados ao CRASI em sua maioria através do sistema de regulação, advindos de encaminhamento da Atenção Primária à Saúde, para determinar o diagnóstico clínico ou adequar a conduta terapêutica. Não houve restrição para o ingresso desses pacientes no serviço, porém, devido à organização do setor, os pacientes, em sua maioria, possuíam demência ou outro déficit cognitivo a esclarecer.

Foram adotados os seguintes critérios de inclusão: idade igual ou superior a 60 anos; ter sido submetido à escala de avaliação clínica da demência (*Clinical Dementia Rating* -CDR)^(10)^; e ter passado pela triagem multidisciplinar, incluindo a triagem fonoaudiológica. Em relação aos critérios de exclusão, foram levados em consideração dados ausentes na ficha de avaliação que comprometeram a interpretação e análise do estudo; prontuários que por algum motivo não puderam ser recuperados no setor de arquivo; indivíduos não brasileiros; indivíduos com comprometimento cognitivo leve (escore no CDR = 0,5); indivíduos que realizaram cirurgias em cabeça e pescoço; e indivíduos que realizaram tratamento fonoaudiológico anterior.

Os participantes foram estratificados em grupos, conforme classificação do grau da demência pelo CDR. O grupo neurotípico (CDR 0) foi composto por idosos sem alterações neurológicas ou psiquiátricas, com pontuação no CDR igual a 0. Já os indivíduos com demência possuíam o diagnóstico médico segundo os critérios de NINCDS-ARDRA (National Institute of Neurological and Communicative Diseases and Stroke - Alzheimer’s Disease and Related Disorders Association)^(2)^ e escore no CDR igual a 1 (demência leve) (CDR 1), 2 (demência moderada) (CDR 2) ou 3 (demência grave) (CDR 3). O Quadro 1 apresenta as principais manifestações cognitivas e funcionais de cada estágio da demência.

**Quadro 1:** Caracterização das fases da demência, de acordo com o Clinical Dementia Rating (CDR), conforme as manifestações cognitivas e funcionais^(2)^

Função	Nível de comprometimento funcional				
	Nenhum (CDR 0)	Questionável (CDR 0,5)	Leve (CDR 1)	Moderado (CDR 2)	Grave (CDR 3)
Memória	Sem comprometimento ou perda leve e inconstante.	Esquecimento leve constante, recorda-se parcialmente de eventos.	Perda moderada de memória para eventos recentes, o que interfere nas atividades de vida diária.	Perda grave de memória. Apenas assunto altamente aprendido continua preservado.	Perda grave de memória. Apenas resquícios de memória estão preservados.
Orientação	Completamente orientado.	Completamente orientado, com leve dificuldade com o tempo.	Dificuldade moderada em relação ao tempo. Orientado no espaço em áreas conhecidas.	Dificuldades graves em relação ao tempo. Frequentemente desorientado no espaço.	Orientado somente em relação a pessoas.
Julgamento e resolução de problemas	Resolve problemas diários e lida bem com negócios e finanças. Julgamento preservado.	Comprometimento leve na resolução de problemas, similaridades e diferenças.	Dificuldade moderada na resolução de problemas, similaridades e diferenças.	Dificuldade grave na resolução de problemas, similaridades e diferenças.	Completamente incapaz de resolver problemas ou realizar julgamentos.
Relações comunitárias	Independente para os níveis usuais de trabalho, compras e participação em grupos sociais.	Comprometimento leve.	Não é independente nestas atividades, embora possa apresentar-se normal no contato casual.	Completamente incapaz de funcionar adequadamente fora do domicílio, apesar de não aparentar.	Completamente incapaz de funcionar adequadamente fora do domicílio
Tarefas domésticas	Tarefas domésticas, passatempos e interesses intelectuais preservados.	Comprometimento leve.	Leve prejuízo em tarefas domésticas. Tarefas mais complexas, passatempos e interesses abandonados.	Apenas tarefas simples estão preservadas. Interesses muito restritos.	Completamente incapaz.
Cuidados pessoais	Independente para cuidar-se.		Necessita de ajuda.	Requer assistência para vestir-se, higiene pessoal e autocuidado.	Totalmente dependente para o autocuidado. Presença de incontinência.

Fonte: adaptado de Macedo Montaño et al.^(10)^

O acolhimento realizado no serviço levava de 30 a 50 minutos, contava com uma equipe multidisciplinar (enfermagem, fisioterapia, fonoaudiologia, geriatria, odontologia e serviço social), na qual, cada categoria profissional realizava uma abordagem inicial, levantando as demandas específicas da área. Este acolhimento ocorria sete ou 15 dias após a primeira consulta com o geriatra. Após passar por todas as especialidades do serviço o idoso era liberado, com as orientações necessárias e os profissionais se dirigiam para a reunião de equipe para discussão dos casos, elaboração do projeto terapêutico singular (PTS) e encaminhamentos a outros serviços, quando necessário.

Para coleta dos dados deste estudo, foram utilizadas informações extraídas dos prontuários físicos dos pacientes: avaliação geriátrica ampla (AGA), que forneceu dados de identificação autorrelatados pelo paciente e/ou cuidador e avaliação funcional e cognitiva, e triagem fonoaudiológica, que forneceu dados da avaliação clínica da deglutição. Os dados de identificação abrangem informações sociodemográficas, como sexo, idade, escolaridade em anos de estudo, e o desempenho em testes de avaliação do estado funcional, cognitivo e emocional. Estes testes incluem o Mini Exame do Estado Mental (MEEM), *Clinical Dementia Rating* (CDR), Escala de Depressão Geriátrica reduzida (GDS) para idosos responsivos, Escala Cornell, Índice de Pfeffer, Escala de Katz, Fluência Verbal Semântica (FVS) e Teste do Desenho do Relógio (TDR), conforme preconizado pela Academia Brasileira de Neurologia (ABN)^(1)^ (Apêndice 1).

Neste estudo, foi examinada a associação entre o desempenho nos instrumentos de avaliação clínica, funcional e de humor quanto à gravidade da demência, mensurada pela Escala para Estadiamento Clínico da Demência (CDR). Não foi realizada correlação entre estes instrumentos e os itens específicos da avaliação clínica da deglutição (Apêndice 2).

Os dados sobre a avaliação clínica da deglutição foram retirados do protocolo de triagem fonoaudiológica (Apêndice 3), uma versão adaptada da Avaliação Clínica da Deglutição, disponível no I Consenso Brasileiro de Nutrição e Disfagia em Idosos Hospitalizados^(11)^ e do Protocolo Fonoaudiológico de Avaliação do Risco para Disfagia (PARD)^(12)^. Durante a avaliação, foram oferecidos 200ml de água em temperatura ambiente, servidos em copos descartáveis padrão, para a avaliação da consistência líquida. Para a consistência néctar, foram disponibilizados 100ml de líquido espessado de acordo com as instruções contidas no rótulo do produto, utilizando o espessante da marca Nestlé *Resource® ThickenUp Clear*, onde a dosagem de 1,2g do espessante foi adicionada para cada 100 ml de água em temperatura ambiente. Já para a consistência sólida, foi oferecida uma unidade de biscoito "Maizena". Essas consistências foram oferecidas na seguinte ordem: néctar, sólido e líquido, seguindo o protocolo utilizado no estudo. Foram verificados os seguintes itens^(12)^: Resíduo oral; Captação alterada do utensílio; Tempo de trânsito oral alterado; Mastigação alterada; Excursão laríngea alterada; Refluxo nasal; Engasgo; Pigarro; Tosse; Voz molhada; Ausculta cervical positiva; Queda na saturação de oxigênio (queda acima de 4%). Para realizar esta classificação, foram adotadas as diretrizes encontradas na literatura especializada, alinhadas com o Protocolo de Avaliação de Risco para Disfagia (PARD)^(12)^, que emprega uma abordagem dicotômica para cada item avaliado, classificando-os como ausentes ou presentes de acordo com os sinais clínicos de disfagia.

A avaliação clínica da deglutição constituía uma prática habitual no serviço fonoaudiológico, na qual estagiários devidamente capacitados conduziam o procedimento, sob a supervisão das fonoaudiólogas responsáveis. Antes de iniciar a avaliação, os avaliadores realizavam a monitorização dos sinais vitais dos pacientes, avaliando a saturação de oxigênio, a frequência cardíaca e a respiratória. Adicionalmente, procediam à ausculta cervical com o intuito de identificar potenciais ruídos laríngeos indicativos de penetração ou aspiração laringotraqueal. Durante todo o processo da avaliação, um oxímetro era empregado para detectar possíveis episódios de broncoespasmo associados à aspiração laringotraqueal, evidenciados pela diminuição na perfusão de oxigênio. Como procedimento padrão do serviço, os casos eram submetidos a discussão após a avaliação clínica, resultando na definição do diagnóstico clínico da deglutição. A classificação da disfagia neste estudo foi realizada com base na proposta do PARD^(12)^, que oferece uma estrutura padronizada para a avaliação e categorização dos distúrbios de deglutição. O PARD utiliza a Escala de Gravidade e Resultados da Disfagia e a Escala de Gravidade da Disfagia para definir sete níveis distintos, cada um refletindo um grau específico de comprometimento e orientando/recomendando condutas terapêuticas. Para garantir clareza e precisão na categorização, este estudo realizou uma adaptação no protocolo: a não utilização das classificações intermediárias (leve a moderada; moderada a grave), cuja presença pode ser associada a fatores de confusão^(13)^. Os critérios detalhados para a classificação da disfagia são apresentados na Figura 1.

**Figura 1:** Classificação funcional da deglutição^(12)^

Fonte: Adaptado de Padovani et al.^(12)^

### Análise dos dados

Os dados foram analisados de forma descritiva e inferencial utilizando-se o software SPSS 25.0. Foi considerado um nível de significância de 5% para as análises inferenciais. Na análise descritiva das variáveis quantitativas, foram calculadas as medidas de tendência central (média) e variabilidade (desvio-padrão). Na análise descritiva das variáveis qualitativas foram calculadas a frequência absoluta e a frequência relativa percentual.

As variáveis quantitativas foram submetidas a análise de normalidade com o Teste Kolmogorov-Smirnov. Diferenças entre médias de dados foram testadas utilizando-se os testes t de Student para amostras independentes (t) e seu correspondente não-paramétrico, o teste de Mann-Whitney. Quando os dois testes revelaram resultados similares mostramos os resultados paramétricos, porém quando houve divergência, mostramos os resultados não-paramétricos.

A caracterização da amostra foi analisada utilizando todos os grupos de CDR com teste de Qui-quadrado (X2) (sem a correção de Yates), ou o teste exato de Fisher (caso a tabela de contingência tivesse apresentado algum valor esperado inferior a cinco) para as variáveis categóricas. Para as variáveis contínuas, foi utilizado o Teste de Análise de Variância – ANOVA e a comparação entre os pares de médias foi feita com o teste de Tukey (*post hoc*).

As variáveis dependentes foram compostas por cada item de avaliação clínica da deglutição, quanto às consistências alimentares, bem como pela classificação funcional da deglutição. Foi feita a análise inferencial de associação destas em relação à gravidade da demência (CDR), utilizando novamente o teste Qui-quadrado ou o teste exato de Fisher quando necessário, com variáveis binárias, para permitir a captação da diferença do valor esperado entre as categorias do CDR.

## RESULTADOS

A amostra inicial do estudo consistia em 230 indivíduos. Destes, 81 foram excluídos por diferentes razões, sendo 52 por apresentarem CDR = 0,5, 1 por ter idade inferior a 60 anos e 28 devido à impossibilidade de recuperar prontuários.

Conforme demonstra a Tabela 1, a amostra foi composta por 102 participantes com demência (68,4%) e 47 neurotípicos (31,5%). Em todos os grupos houve predomínio do sexo feminino. A idade variou entre 60 e 92 anos, com média de 74 anos em idosos neurotípicos e 77 em idosos com diagnóstico de demência. Quanto à escolaridade verificou-se que idosos neurotípicos apresentavam maior média de anos em comparação aos idosos com demência.

**Tabela 1:** Caracterização da amostra conforme as variáveis sociodemográficas, avaliação funcional, cognitiva e de humor, e tipo de demência em idosos, conforme o CDR e suas associações

		**CDR 0**	**CDR 1**	**CDR 2**	**CDR 3**	**Total**	**p**
**Quantidade total de indivíduos (n/%)**		**47 (31,3%)**	**37 (24,6%)**	**40 (26,6%)**	**25 (17,3%)**		
Idade em anos (Média ± DP)		74,49 ± 6,92	75,70 ± 8,42	77,03 ± 7,83	79,80 ± 5,74	-	0,033*
Escolaridade em anos (Média ± DP)		7,38 ± 6,05	4,43 ± 4,55	7,05 ± 5,35	6,60 ± 5,29	-	0,074
Sexo feminino (n/%)		36 (76,6%)	29 (78,4%)	22 (55,0%)	18 (72%)	105 (70,9%)	0,086
Tipo de demência (n/%)							<0,001*
Alzheimer		-	5 (13,5%)	10 (25,0%)	12 (48%)	27 (26,4%)	
Vascular		-	10 (27%)	9 (22,5%)	11 (44%)	30 (29,4%)	
Mista		-	2 (5,4%)	7 (17,5%)	0 (0%)	9 (8,82%)	
Em investigação		-	19 (51,4%)	14 (35,0%)	2 (8%)	35 (34,3%)	
Outros*		-	1 (2.7%)	0 (0%)	0 (0%)	1 (0,98%)	
**Instrumentos de avaliação cognitiva, funcional e de humor**							
MEEM	Alterado	24 (51,1%)	35 (94,6%)	40 (100%)	23 (100%)	132 (89,7%)	<0,001*
	Total	47	37	40	23	147	
FVS (animais)	Alterado	14 (31,8%)	21 (56,8%)	28 (75,7%)	18 (100%)	81 (60,4%)	<0,001*
	Total	44	35	37	18	134	
TDR	Alterado	16 (38,1%)	18 (62,1%)	33 (82,5%)	14 (100%)	81 (66,9%)	<0,001*
	Total	42	29	36	14	121	
Pfeffer	Alterado	7 (15,2%)	34 (97,1%)	37 (97,4%)	19 (95%)	97 (69,7%)	<0,001*
	Total	46	35	38	20	139	
Katz	D	3 (6,4%)	5 (13,9%)	12 (31,6%)	17 (73,9%)	37 (25,6%)	<0,001*
	P.D.	3 (6,4%)	5 (13,9%)	18 (47,4%)	5 (21,7%)	31 (21,5%)	
	I.	41 (87,2%)	26 (72,2%)	8 (21,1%)	1 (4,3%)	76 (52,7%)	
	Total	47	36	38	23	144	
Depressão	Sim	20 (43,5%)	22 (64,7%)	24 (64,9%)	7 (58,3%)	73 (56,5%)	0,159
	Total	46	34	37	12	129	

* Significância estatística (p<0,005)

**Legenda:** DP = desvio-padrão; n=frequência absoluta; %=frequência relativa; MEEM = Mini-Exame do Estado Mental; FVS = Fluência Verbal Semântica; TDR = Teste do Desenho do Relógio; D = Dependente; PD = Parcialmente Dependente; I = Independente

A análise da classificação da demência indicou que a maioria dos indivíduos no grupo CDR 1 ainda não havia recebido um diagnóstico conclusivo (51,4%), com o tipo de demência em processo de investigação, seguido da demência vascular (27%), Alzheimer (13,5%), demência mista (5,4%) e, por fim, um caso de afasia progressiva primária (2,7%), variante da demência frontotemporal. Da mesma forma, no grupo CDR 2, predominaram os casos sem diagnóstico conclusivo (35%), seguidos por Alzheimer (25%), demência vascular (22,5%) e demência mista (17,5%). Por outro lado, no grupo CDR 3, cerca da metade dos indivíduos foi diagnosticada com Alzheimer (48%), seguido de demência vascular (44%), enquanto apenas dois estavam sem diagnóstico concluído (8%).

Ainda de acordo com a Tabela 1, é possível verificar que a análise dos instrumentos de avaliação funcional e cognitiva evidenciou que as alterações começaram a se manifestar desde a fase leve da demência. Observou-se um aumento progressivo nas taxas de alteração à medida que a demência avançava. No grupo CDR 2, o percentual de alteração no MEEM atingiu 100%, indicando um comprometimento generalizado da função cognitiva. No entanto, é importante destacar que também foram observadas prevalências significativas de alteração nos indivíduos neurotípicos. Além disso, tanto o teste de Fluência Verbal Semântica (FVS) quanto o Teste do Desenho do Relógio (TDR) apresentaram uma prevalência de alteração já na fase leve da demência, com taxas de alteração atingindo 100% no grupo CDR 3, evidenciando uma deterioração cognitiva progressiva ao longo da evolução da demência.

A Tabela 2 apresenta os resultados da avaliação clínica da deglutição nos quatro grupos e apresenta quais sinais clínicos de alteração da deglutição apresentaram diferença estatística significativa.

**Tabela 2:** Resultado da análise descritiva das variáveis da avaliação clínica da deglutição em relação ao CDR, conforme consistências alimentares

**Item avaliado/ Consistência avaliada**			**CDR**				
			**0**	**1**	**2**	**3**	**Frequência geral (n/%)**
Escape oral anterior	Néctar	Presente	2 (4,5%)	2 (5,6%)	1 (2,6%)	4 (17,4%)	9 (6,3%)
		Total	44	36	38	23	
	Sólido	Presente	2 (4,4%)	2 (5,7%)	4 (10%)	1 (5%)	9 (6,4%)
		Total	45	35	40	20	
	Líquido **^c,e^**	Presente	3 (7,1%)	5 (14,3%)	3 (7,9%)	7 (31,8%)	18 (13,1%)
		Total	42	35	38	22	
Resíduo oral	Néctar **^c,e^**	Presente	0 (0%)	1 (2,8%)	0 (0%)	4 (18,2%)	4 (2,8%)
		Total	44	36	37	23	
	Sólido **^a^**	Presente	6 (13,6%)	12 (34,3%)	11 (28,9%)	7 (38,9%)	36 (26,6%)
		Total	44	35	38	18	
	Líquido **^c,e,f^**	Presente	0 (0%)	0 (0%)	0 (0%)	4 (18,2%)	4 (3%)
		Total	42	35	39	22	
Captação alterada do utensílio	Néctar	Presente	3 (7%)	8 (22,2%)	9 (24,3%)	3 (13%)	23 (16,5%)
		Total	43	36	37	23	
	Sólido	Presente	7 (15,9%)	7 (20%)	11 (28,2%)	4 (20%)	29 (21%)
		Total	44	35	39	20	
	Líquido	Presente	4 (9,5%)	7 (20%)	7 (18,4%)	6 (27,3%)	24 (17,5%)
		Total	42	35	38	22	
Tempo de trânsito oral alterado	Néctar	Presente	3 (6,8%)	8 (22,9%)	8 (21,1%)	4 (18,2%)	23 (16,5%)
		Total	44	35	38	22	
	Sólido	Presente	4 (8,9%)	8 (22,9%)	9 (23,7%)	5 (26,3%)	26 (18,9%)
		Total	45	35	38	19	
	Líquido **^c^**	Presente	4 (9,8%)	6 (17,1%)	6 (16,2%)	9 (40,9%)	25 (18,5%)
		Total	41	35	37	22	
Mastigação alterada	Sólido	Presente	13 (29,5%)	16 (45,7%)	19 (48,7%)	8 (40%)	44 (31,8%)
		Total	44	35	39	20	
Excursão laríngea reduzida	Néctar**^b^**	Presente	4 (9,3%)	7 (19,4%)	10(27%)	3(13,6%)	24 (17,3%)
		Total	43	36	37	22	
	Sólido	Presente	5 (11,1%)	6 (17,1%)	9 (23,1%)	5 (25%)	25 (17,9%)
		Total	45	35	39	20	
	Líquido	Presente	6 (14,6%)	6 (17,1%)	8 (21,8%)	6 (26,1%)	26 (18,7%)
		Total	41	35	38	23	
Refluxo nasal	Néctar	Presente	2 (4,5%)	0 (0%)	0 (0%)	1 (4,5%)	3 (2,1%)
		Total	44	36	37	22	
	Sólido	Presente	2 (4,4%)	0 (0%)	0 (0%)	0 (0%)	2 (1,4%)
		Total	45	35	40	20	
	Líquido	Presente	2 (4,8%)	1 (2,9%)	0 (0%)	1 (4,3%)	4 (2,8%)
		Total	42	35	39	23	
Engasgo	Néctar	Presente	2 (4,4%)	0 (0%)	1 (2,7%)	2 (8,7%)	5 (3,5%)
		Total	44	36	37	23	
	Sólido	Presente	2 (4,4%)	0 (0%)	0 (0%)	1 (5%)	3 (2,1%)
		Total	45	35	40	20	
	Líquido	Presente	3 (7,1%)	1 (2,9%)	3 (7,7%)	4 (17,4%)	11 (7,9%)
		Total	42	35	39	23	
Pigarro	Néctar	Presente	4 (9,1%)	4 (11,1%)	7 (18,9%)	4 (17,4%)	19 (13,5%)
		Total	44	36	37	23	
	Sólido	Presente	4 (8,9%)	4 (11,4%)	9 (22,5%)	2 (10%)	19 (13,5%)
		Total	45	35	40	20	
	Líquido **^b^**	Presente	2 (4,9%)	4 (11,4%)	8 (21,1%)	4 (17,4%)	18 (13,1%)
		Total	41	35	38	23	
Tosse	Néctar	Presente	2 (4,5%)	0 (0%)	2 (5,4%)	1 (4,3%)	5 (3,5%)
		Total	44	36	37	23	
	Sólido **^f^**	Presente	2 (4,4%)	0 (0%)	3 (7,9%)	3 (15%)	8 (5,7%)
		Total	45	35	38	20	
	Líquido ^b^	Presente	2 (4,8%)	2 (5,7%)	8 (21,1%)	4 (17,4%)	16 (11,5%)
		Total	42	35	38	23	
Voz molhada	Néctar	Presente	10 (23,3%)	13 (36,1%)	6 (16,2%)	5 (23,8%)	34 (24,8%)
		Total	43	36	37	21	
	Sólido	Presente	9 (20%)	7 (20%)	7 (17,9%)	2 (10,5%)	25 (18,1%)
		Total	45	35	39	19	
	Líquido	Presente	9 (21,4%)	13 (37,1%)	11 (28,9%)	6 (31,6%)	39 (29,1%)
		Total	42	35	38	19	
Ausculta cervical positiva	Néctar **^b,c,d^**	Presente	2 (4,5%)	3 (8,3%)	12 (32,4%)	5 (23,8%)	25 (18,1%)
		Total	44	36	37	21	
	Sólido	Presente	5 (11,1%)	6 (17,1%)	6 (15,4%)	5 (27,8%)	22 (15,9%)
		Total	45	35	39	18	
	Líquido **^b,c^**	Presente	2 (4,8%)	5 (14,3%)	12 (31,6%)	7 (31,8%)	26 (18,9%)
		Total	42	35	38	22	
Queda SPO2	Néctar	Presente	3 (7,7%)	1 (3,3%)	1 (2,9%)	1 (5,9%)	7 (5,8%)
		Total	39	30	34	17	
	Sólido	Presente	2 (5,1%)	1 (3,3%)	1 (3%)	2 (13,3%)	6 (5,1%)
		Total	39	30	33	15	
	Líquido **^c,f^**	Presente	0 (0%)	0 (0%)	2 (6,1%)	3 (15,8%)	5 (4,1%)
		Total	38	30	33	19	

Teste Qui-Quadrado de Pearsonquadrado

**Legenda:** n=frequência absoluta; %=frequência relativa; a= resultado estatisticamente significante entre CDR 0 e CDR 1; b= resultado estatisticamente significante entre CDR 0 e CDR 2; c=resultado estatisticamente significante entre CDR 0 e CDR 3; d= resultado estatisticamente significante entre CDR 1 e CDR 2; e= resultado estatisticamente significante entre CDR 2 e CDR 3; f= resultado estatisticamente significante entre CDR 1 e CDR 3

A maioria das variáveis em que ocorreu diferença estatística significativa mostrou um maior alteração do CDR 3 em relação ao CDR 0: escape oral para líquido (p = 0,012), resíduo oral para néctar (p = 0,010), resíduo oral para líquido (p = 0,010), tempo de trânsito oral alterado para líquido (p = 0,015), ausculta cervical positiva para néctar (p= 0,026), ausculta cervical positiva para líquidos (p= 0,013) e queda na saturação de oxigênio para líquidos (p = 0,029). Foi encontrada maior alteração do CDR 3 em comparação ao CDR 1 nas variáveis: resíduo oral para líquido (p = 0,016), tosse para sólido (p =0,039), queda na saturação de oxigênio para líquido (p= 0,047).

Apenas o resíduo oral para sólido mostrou maior alteração no grupo CDR 1 quando comparado ao CDR 0 (p = 0,030).

Foi observada diferença estatística significativa entre CDR 0 e CDR 2, com maior alteração no CDR 2 em: excursão laríngea reduzida para néctar (p =0,044), pigarro para líquido (p = 0,043), tosse para líquido (p = 0,041), ausculta cervical positiva para néctar (p = 0,001) e ausculta cervical para líquido (p = 0,002).

Com relação à diferença estatística entre CDR 1 e CDR 2, a ausculta cervical positiva para néctar apresentou diferença estatística significativa, com maior alteração no CDR 2 (p = 0,019).

Ao comparar CDR 2 e CDR 3 verificou-se diferença estatística significativa com maior alteração no CDR 3 em: escape oral para líquido (p = 0,026), resíduo oral para néctar (p = 0,016), resíduo oral para líquido (p = 0,012).

Comparando CDR 1 e CDR 3 verificou-se diferença estatística com maior alteração no CDR 3 em: resíduo oral para líquido (p = 0,016), tosse para sólido (p =0,039), queda na saturação de oxigênio para líquido (p= 0,047).

Conforme aponta a Tabela 3, o resultado da classificação funcional da deglutição, demonstrou diferença estatística entre a deglutição normal, disfagia moderada e disfagia grave entre os grupos. A deglutição funcional (p= 0,520) e disfagia leve (0,103) foram estatisticamente equivalentes entre os grupos, porém na análise grupo a grupo houve diferença estatística entre o CDR 0 e CDR 2 em relação à presença de disfagia leve.

**Tabela 3:** Resultado da análise descritiva da classificação funcional da deglutição com a variável de estadiamento da demência

	CDR				
	0 (N=41)	1 (N= 31)	2 (N = 35)	3 (N= 23)	Total
	n (%)	n (%)	n (%)	n (%)	n (%)
Deglutição normal **^a,b,c^**	30 (73,2%)	13 (41,9%)	13 (37,1%)	6 (26,1%)	62 (48%)
Deglutição funcional	9 (22,0%)	3 (9,7%)	5 (14,3%)	3 (13,0%)	20 (15,5%)
Disfagia leve **^b^**	1 (2,4%)	4 (12,9%)	7 (20,0%)	4 (17,4%)	16 (12,4%)
Disfagia moderada **^a,b^**	0 (0%)	8 (25,8%)	10 (28,6%)	2 (8,7%)	20 (15,5%)
Disfagia grave **^c,e,f^**	1 (2,4%)	2 (6,5%)	0 (0%)	8 (34,8%)	11 (8,5%)

Qui-Quadrado de Pearson

**Legenda:** N= número de participantes por grupo conforme CDR; n=frequência absoluta; %=frequência relativa; a= resultado estatisticamente significante entre CDR 0 e CDR 1; b=resultado estatisticamente significante entre CDR 0 e CDR 2; c= resultado estatisticamente significante entre CDR 0 e CDR 3; d= resultado estatisticamente significante entre CDR 1 e CDR 2; e= resultado estatisticamente significante entre CDR 2 e CDR 3; f= resultado estatisticamente significante entre CDR 1 e CDR 3.

Na comparação grupo a grupo, verificou-se que houve diferença estatística significativa entre CDR 0 e os demais grupos, no qual o CDR 0 associou-se a ter deglutição normal e os demais grupos não: CDR 1 (p = 0,007), CDR 2 (p= 0,002) e CDR 3 (p <0,001).

O CDR 1 associou-se à presença de disfagia leve quando comparado ao CDR 0 (p = 0,021). O CDR 2 associou-se a presença de disfagia leve e o CDR 0 à ausência (p = 0,021). Associou-se também à presença de disfagia moderada e o CDR 0 à ausência (p < 0,001).

O CDR 3 associou-se à presença de disfagia grave quando comparado ao CDR 0 (p < 0,001), ao CDR 1 (p = 0,011) e ao CDR 2 (p < 0,001).

## DISCUSSÃO

O principal achado deste estudo foi a presença de disfagia em todas as fases da demência. Há uma heterogeneidade nos estudos que abordam a deglutição em idosos com demência. Alguns focaram na caracterização dos desafios alimentares em indivíduos com doença de Alzheimer^(14)^, enquanto outros buscaram avaliar o risco de disfagia associado à doença de Alzheimer^(15)^. Certas pesquisas trataram da prevalência da disfagia em contextos demenciais^(16)^, e outras utilizaram medidas objetivas para a avaliação da deglutição^(17-19)^.

Estudos com pacientes portadores da Doença de Alzheimer são mais prevalentes, dada a sua natureza como o tipo mais comum de demência. Poucas pesquisas investigaram a deglutição abrangendo todas as fases da demência e apresentaram os resultados de forma estratificada de acordo com o estágio da doença^(8,16,20)^. Considerando a necessidade de uma abordagem mais específica da deglutição nas fases leve, moderada e grave da demência, propusemo-nos a caracterizar essas alterações conforme os estágios da demência. Além disso, um grupo composto por idosos neurotípicos foi estabelecido para comparação com idosos com demência, seguindo a abordagem de alguns estudos anteriores^(14,15,21)^.

### Características sociodemográficas e tipo de demência

Neste estudo, observou-se uma predominância do sexo feminino na amostra, fenômeno atribuível à feminização do envelhecimento e à maior expectativa de vida das mulheres, que vivem aproximadamente 8 anos a mais que os homens^(22)^. Embora não tenha sido identificada diferença estatística significativa por sexo nas distintas fases da demência, a literatura relata uma maior prevalência de demência em mulheres em todas as fases da doença^(8,14,16,18-20)^.

Quanto à faixa etária, constatou-se que os indivíduos com demência eram mais avançados em idade em comparação com os indivíduos neurotípicos. As correlações estatísticas estabelecem que a idade dos indivíduos aumentou à medida que a demência progrediu. Esse resultado era esperado, visto que a demência é uma síndrome predominante em indivíduos idosos, e pessoas mais velhas têm uma probabilidade mais elevada de desenvolver demência^(22)^. Adicionalmente, a média de idade dos participantes com demência foi superior à média de idade dos participantes neurotípicos, uma observação alinhada com resultados de estudos semelhantes^(14,21)^.

No que tange à escolaridade, não foram observadas diferenças em relação ao estágio da demência. Entretanto, a literatura destaca que a baixa escolaridade representa um fator preditivo para o desenvolvimento da demência, uma vez que níveis adequados de instrução contribuem para o aumento da densidade sináptica e compensação de possíveis déficits intelectuais^(2)^. É importante notar que a avaliação da deglutição utilizada neste estudo não está vinculada ao nível educacional dos participantes, pois não exigiu compreensão de comandos verbais complexos ou escritos para a alimentação.

No que diz respeito à prevalência dos tipos de demência, verificou-se que a Demência Vascular (DV) foi a mais comum na amostra, representando 29,4%, seguida pela Doença de Alzheimer (DA) com 26,4%, a demência mista com 8,82%, e, por fim, outros tipos de demência com 0,98%. É relevante destacar que, neste estudo, a categoria "outros" foi utilizada devido à baixa incidência desse tipo na amostra, sendo que apenas um indivíduo apresentou afasia progressiva primária. Esses resultados contrastam com estudos epidemiológicos e clínicos que apontam para uma maior prevalência de DA, seguida pela DV^(2,3)^.

Estudos que exploram a relação entre demência e deglutição também apresentam resultados discrepantes em comparação com as descobertas deste estudo. Em uma pesquisa conduzida com idosos com demência^(19)^ foi relatado que 54,4% da amostra apresentava Doença de Alzheimer (DA), com apenas 20,6% diagnosticados com Demência Vascular (DV). Além disso, 22,9% dos participantes manifestaram demência associada ao Parkinson, uma condição que não foi observada na presente pesquisa. Por outro lado, um estudo adicional identificou uma frequência mais elevada de DA (52,9%) e demência mista (25,1%), com apenas 3,1% de casos de DV^(8)^.

É essencial destacar que, no nosso estudo, a maioria dos participantes diagnosticados com demência ainda estava em fase de investigação médica para determinar o tipo específico.

### Aspectos funcionais, cognitivos e de humor

O CDR possibilita uma avaliação abrangente do indivíduo. Dessa forma, os demais instrumentos de rastreamento cognitivo, avaliação funcional e de humor foram empregados exclusivamente para a caracterização da amostra neste estudo. Não foram conduzidas correlações entre o desempenho nessas ferramentas e os itens da avaliação clínica da deglutição.

A maioria dos participantes na amostra foi constituída por pacientes com demência, destacando-se uma predominância da fase moderada (26,6%), seguida pela fase leve (24,6%) e, por último, a demência grave (17,3%). Embora os resultados da literatura sobre a distribuição das fases da demência sejam heterogêneos, nossos achados quanto a essa distribuição assemelham-se a outros estudos previamente conduzidos^(16,18)^.

No que diz respeito ao Mini-Exame do Estado Mental, neste estudo, as pontuações de corte foram ajustadas de acordo com o nível de escolaridade. Esses ajustes também foram aplicados aos demais instrumentos cognitivos e funcionais nesta amostra, levando em consideração a diversidade sociocultural e educacional da população brasileira^(1,22)^. A adaptação baseada na escolaridade teve como objetivo evitar resultados falso-positivos e falso-negativos durante o processo de avaliação.

Neste estudo, constatou-se uma correlação estatisticamente significativa entre o desempenho nas avaliações funcional e cognitiva e a gravidade da demência, corroborando achados presentes na literatura^(2)^. Houve uma prevalência de alterações nos grupos com demência no MEEM, FVS (animais), TDR e Pfeffer, indicando que a maioria da amostra era composta por idosos com menor funcionalidade e maior dependência.

### Avaliação clínica da deglutição

Neste estudo, a detecção de alterações na deglutição foi realizada por meio de um protocolo adaptado de avaliação clínica, no qual foram observados diversos itens durante a ingestão de diferentes consistências alimentares.

A avaliação clínica da deglutição envolve a observação de sinais clínicos indicativos de disfagia e é frequentemente empregada, especialmente em ambientes hospitalares. Vale ressaltar que esta abordagem é não invasiva, de fácil reprodução e possui baixo custo^(23)^. Embora esses méritos sejam reconhecidos, a literatura discute de forma limitada a eficácia da avaliação clínica, considerando os métodos instrumentais, como a videofluoroscopia e a videoendoscopia da deglutição, como os mais confiáveis para identificar alterações na deglutição^(24)^. Entretanto, em situações específicas, como em casos de demência, a realização de exames instrumentais pode ser desafiadora, uma vez que tais avaliações demandam níveis adequados de atenção, preservação da compreensão oral e cooperação por parte do paciente^(5)^.

Neste estudo, observou-se que os idosos com demência leve apresentaram uma frequência mais elevada de resíduo sólido na cavidade oral em comparação com os idosos neurotípicos. Essa descoberta pode ser explicada pela diminuição da força da língua, juntamente com a redução da sensibilidade na cavidade oral, na região faríngea e comprometimento na coordenação motora oral, características associadas aos casos de demência, conforme relatado na literatura^(25)^. Não foram identificadas disparidades neste componente específico da avaliação clínica nas outras fases da demência. Uma explicação plausível para esse padrão é a observação de que a restrição alimentar em relação a alimentos sólidos é comumente relatada em indivíduos que estão há mais tempo enfrentando a doença, estando frequentemente associada à ocorrência de engasgos, dificuldades na deglutição e à deterioração da saúde oral^(26)^ .

Os resultados deste estudo assemelham-se aos achados de uma pesquisa envolvendo 26 idosas com doença de Alzheimer nos estágios leve e moderado, na qual foi identificada a presença de resíduo oral em consistências pastosa e sólida (34,6%)^(17)^. Contudo, a comparação direta entre os estudos é limitada devido às diferenças na estratificação das fases, nos tipos de demência e nas abordagens das consistências utilizadas.

Na fase moderada da demência, observou-se uma redução na excursão laríngea durante a deglutição de consistência néctar, ocorrências de pigarro e tosse com líquidos, além de ausculta cervical positiva para néctar e líquidos em comparação com indivíduos neurotípicos. A alteração identificada em nossa pesquisa pode estar associada à maior viscosidade do néctar em comparação com o líquido, exigindo, consequentemente, uma maior força durante o ato de deglutição^(27)^.

A observação de uma maior força necessária para deglutir néctar em comparação com líquidos mais finos destaca um aspecto relevante no contexto da disfagia associada à demência, particularmente na fase moderada. A consistência do néctar, por sua natureza mais espessa, exige uma resposta muscular mais intensa durante a deglutição. Essa demanda adicional de força pode ser atribuída à necessidade de superar a viscosidade do néctar, tornando a movimentação do bolo alimentar mais desafiadora^(27)^.

Ao longo do processo de envelhecimento, ocorrem modificações significativas em estruturas cruciais para a deglutição, incluindo a diminuição do tônus na musculatura intrínseca e extrínseca da laringe, bem como a ossificação das cartilagens laríngeas, resultando na redução da excursão laríngea^(4,6)^. Em situações de demência associada, essas alterações são exacerbadas^(16,17)^. É relevante ressaltar que o mecanismo de excursão laríngea desempenha um papel fundamental no processo de deglutição, agindo de maneira coordenada com o osso hióide para assegurar a proteção da via aérea, contribuindo para uma deglutição segura e eficiente. Dessa forma, mudanças nesse mecanismo podem estar associadas à ocorrência de broncoaspiração^(6)^.

Neste estudo, a manifestação de tosse ao ingerir líquidos foi associada à presença de demência moderada. A presença de tosse durante ou após a deglutição sugere a possível ocorrência de aspiração ou penetração de alimentos e/ou líquidos, desencadeando sensibilidade laríngea e estimulando o reflexo de proteção das vias aéreas inferiores na tentativa de expelir o conteúdo^(6)^. Em idosos, a queixa de tosse é frequentemente observada, e quando associada a quadros demenciais, evidencia a dificuldade na funcionalidade da deglutição decorrente do declínio cognitivo e do déficit na função motora oral^(8)^.

Na fase grave da demência, observou-se uma série de alterações na deglutição, incluindo prevalência de escape oral anterior com líquido, resíduo oral com néctar e líquido, tempo de trânsito oral aumentado para líquidos, tosse com sólidos, ausculta cervical positiva para néctar e líquido, bem como queda na saturação de oxigênio com líquido.

A presença significativa de escape oral anterior associou-se especificamente à demência grave, embora também tenha sido identificada em outras fases da demência. Em um estudo anterior, que utilizou a videofluoroscopia em sujeitos com diferentes tipos de demência nas fases moderada e grave, foram relatadas alterações relacionadas ao escape oral em 24% da amostra^(19)^.

Em outro estudo, a associação entre escape oral anterior e demência grave foi explicada pela presença de apatia em estágios mais avançados da demência. A presença de apatia pode influenciar negativamente na velocidade das fases antecipatória e preparatória oral da deglutição, o que, por sua vez, facilita o escape de alimentos da boca^(5)^.

O tempo de trânsito oral (TTO) alterado associou-se à demência grave, indicando uma relação proporcional entre a evolução do *Clinical Dementia Rating* (CDR) e o TTO. Uma pesquisa anterior com idosos diagnosticados com doença de Alzheimer (DA) corroborou esses achados, evidenciando um significativo aumento no TTO durante a demência grave^(20)^. O prolongamento desse tempo pode ser atribuído à presença de agnosia orotátil, disfunções nas funções executivas e à diminuição da força da língua. A agnosia orotátil, manifestada pela redução do input sensorial na cavidade oral, dificulta a percepção do alimento durante as fases preparatória e oral da deglutição^(25)^.

A manifestação de tosse nas fases moderada e grave da demência indica o início de episódios de penetração e/ou aspiração laringotraqueal com essas consistências^(15)^. Alguns estudos indicam a adaptação da textura e a alteração de consistência, especialmente o espessamento de líquidos finos, como uma abordagem para promover uma deglutição segura e eficiente em pacientes com disfagia orofaríngea e demência^(8)^. Esses achados possivelmente explicam a ausência de diferença estatística na ocorrência de tosse com néctar neste estudo.

A detecção de ausculta cervical positiva para néctar e líquido em indivíduos com demência grave revela implicações clínicas relevantes, indicando uma possível ligação entre comprometimento cognitivo avançado e disfunções na deglutição^(15)^. Esta observação sugere a presença de aspiração laringotraqueal, elevando o risco de complicações respiratórias, como pneumonia. Na demência grave, os desafios nas fases antecipatória e preparatória da deglutição são notáveis, resultando em uma coordenação ineficaz entre os músculos orofaciais e a laringe^(7)^. A identificação precoce da ausculta cervical positiva sublinha a importância da avaliação clínica da deglutição nessa população.

Apesar de não ter apresentado significância estatística neste estudo, a captação adequada de utensílios durante as refeições é uma habilidade motora fina que pode ser afetada nas demências, prejudicando a autonomia e qualidade de vida^(5)^. Indivíduos com demência podem demonstrar lentidão nos movimentos e falta de destreza nas mãos. Conforme a demência avança, a perda de memória e a redução na capacidade de planejamento podem gerar esquecimento das técnicas corretas para usar utensílios, dificultando a organização dos movimentos necessários para a alimentação^(26)^.

Outro aspecto que, embora não tenha demonstrado diferença estatística entre os grupos, merece destaque é a mastigação. Nos estágios iniciais da demência, são comuns dificuldades na mastigação de sólidos, influenciadas pela deterioração cognitiva que compromete a coordenação entre os músculos da mandíbula e a língua^(26)^. Conforme a demência avança, desafios adicionais surgem, como problemas na identificação e manipulação adequada dos alimentos, resultando em dificuldades mastigatórias e risco aumentado de aspiração^(25,26)^. A adaptação do ambiente alimentar e a modificação da consistência dos alimentos, como a oferta de opções mais macias, tornam-se estratégias essenciais para facilitar a mastigação em indivíduos com demência^(8)^.

Em nosso estudo também não houve associação entre refluxo nasal e demência. Esta relação não é amplamente explorada, não havendo até o momento evidências substanciais que estabeleçam uma ligação direta entre refluxo nasal e demência.

### Classificação funcional da deglutição

Ao examinar a classificação funcional da deglutição (conforme mostrado na Tabela 3), observa-se que os idosos sem comprometimentos neurológicos exibiram uma deglutição dentro da normalidade. Por outro lado, aqueles com demência leve manifestaram uma disfagia moderada, enquanto os idosos com demência moderada apresentaram graus variados de disfagia, indo de leve a moderada. Por fim, os idosos com demência grave evidenciaram um quadro de disfagia grave.

A literatura dedicada ao estudo da deglutição em contextos demenciais destaca uma deterioração na funcionalidade da deglutição e um aumento do risco de disfagia à medida que a demência progride^(9)^. Em um estudo, por meio de uma avaliação clínica, os autores constataram que a prevalência de disfagia em indivíduos com doença de Alzheimer (DA) leve e moderada foi de 16,7%, enquanto na fase grave atingiu 91,8%^(15)^. Outros autores^(21)^ ao investigarem parâmetros eletrofisiológicos da deglutição em todas as fases da DA, concluíram que pacientes com DA apresentavam características eletrofisiológicas indicativas de disfagia, mesmo nos estágios iniciais da doença.

A comparação deste estudo com outros relatados na literatura é limitada, devido às disparidades na amostra e nos instrumentos de avaliação da deglutição. Os resultados e associações são de natureza inferencial, sendo derivados da análise estatística conduzida e das semelhanças identificadas.

O presente estudo apresenta algumas limitações. Primeiramente, a ausência de estudos prévios que tenham empregado o mesmo instrumento de avaliação da deglutição em idosos com demência restringe nossa capacidade de comparação, visto que diferentes métodos de avaliação foram utilizados em estudos anteriores. Além disso, a impossibilidade de analisar separadamente as alterações de deglutição em cada tipo de demência representa outra limitação significativa. Dada a diversidade dos tipos de demência na nossa amostra, a generalização dos resultados para cada categoria específica torna-se desafiadora.

A falta de um instrumento específico e validado para a avaliação clínica da deglutição em idosos com demência é outra limitação relevante. Adicionalmente, uma parte substancial da nossa amostra encontrava-se em processo de investigação, sem um diagnóstico definitivo de demência estabelecido. Isso compromete a caracterização precisa da amostra, limitando a generalização dos resultados para a população de idosos com demência de forma abrangente.

Em conjunto, essas limitações enfatizam a necessidade contínua de pesquisas futuras que busquem superar esses desafios metodológicos e contribuam para uma compreensão mais abrangente e precisa das alterações de deglutição em idosos com demência.

## CONCLUSÃO

Este estudo identificou padrões distintos de funcionalidade na deglutição em diferentes estágios da demência, conforme avaliado pelo CDR. Os aspectos de destaque em cada CDR foram os seguintes:

- CDR 0 (Neurotípico): padrão de deglutição normal;
- CDR 1 (Demência leve): Presença de resíduo oral para sólidos. Associado à disfagia leve;
- CDR 2 (Demência moderada): Excursão laríngea reduzida para néctar, presença de pigarro para líquidos, presença de tosse para líquidos, ausculta cervical positiva para néctar. Associado à disfagia leve e disfagia moderada;
- CDR 3 (Demência grave): Presença de escape oral anterior para líquidos, presença de resíduo oral para néctar e líquidos, tempo de trânsito oral alterado para líquidos, ausculta cervical alterada para néctar e líquidos, presença de tosse para sólidos, queda na saturação de oxigênio para líquidos. Associado à disfagia grave.

Em síntese, este estudo oferece evidências da associação entre a gravidade da demência, avaliada pelo CDR, e a deterioração da função de deglutição. Essas descobertas têm implicações clínicas cruciais para o manejo de pacientes com demência, destacando a importância da avaliação e intervenção precoces na disfagia para melhorar a qualidade de vida e prevenir complicações associadas à alimentação e deglutição neste grupo.

## Apêndice 1 Descrição dos instrumentos de avaliação funcional, cognitiva e de humor, conforme preconizado pela Academia Brasileira de Neurologia^(1,2)^

**Table:**

**Instrumento**	**Descrição**
Mini-Exame do Estado Mental	Instrumento de avaliação cognitiva utilizado para avaliar o estado mental e identificar possíveis déficits cognitivos. Composta por perguntas e tarefas que abrangem diferentes aspectos cognitivos, como orientação temporal e espacial, memória, atenção e linguagem, a pontuação máxima no MEEM é 30 pontos. Escores mais elevados indicam melhor funcionamento cognitivo, enquanto escores mais baixos podem sugerir comprometimento cognitivo. Para uma interpretação mais específica, neste estudo, foram estabelecidos pontos de corte sugeridos com base no nível de escolaridade do indivíduo: 20 pontos para analfabetos, 25 pontos para aqueles com 1 a 4 anos de estudo, 26 pontos para os que possuem 5 a 8 anos de estudo, 28 pontos para 9 a 11 anos de estudo e 29 pontos para aqueles com escolaridade acima de 11 anos.
C*linical Dementia Rating (CDR)*	Ferramenta clínica utilizada para avaliar a gravidade da demência. Essa escala, empregada como instrumento de estadiamento, permite compreender a evolução e a intensidade dos sintomas cognitivos em indivíduos com demência. Ao atribuir pontuações em diversos domínios, incluindo memória, orientação, julgamento e comunicação, o CDR gera uma classificação que varia entre 0 (neurotípico), 0,5 (comprometimento cognitivo leve), 1 (demência leve), 2 (demência moderada) e 3 (demência grave).
Escala de Depressão Geriátrica (GDS)	Instrumento utilizado para avaliar sintomas depressivos em idosos. Composta por uma série de perguntas de resposta binária (sim/não), a GDS visa identificar indicadores de depressão, como desânimo, tristeza e falta de interesse em atividades cotidianas. Pontuações mais elevadas refletem maior gravidade dos sintomas depressivos. A escala varia de 0 a 15, e uma pontuação ≥ 5 sugere a possibilidade de um quadro depressivo.
Escala de Cornell	Instrumento destinado à avaliação de sintomas comportamentais em idosos com demência. Composta por uma série de perguntas sobre comportamentos observados nos últimos sete dias, a escala é respondida por um cuidador ou familiar. Os itens abordam áreas como agressividade, resistência a cuidados, agitação, depressão, alucinações, delírios, entre outros. A pontuação total varia de 0 a 45, sendo que escores mais elevados indicam maior gravidade de sintomas comportamentais
Índice de Pfeffer	ferramenta de avaliação que visa investigar a capacidade de desempenhar atividades instrumentais da vida diária (AIVDs) em idosos. Consiste em uma entrevista aplicada ao cuidador ou familiar do idoso, abordando questões relacionadas à autonomia nas tarefas cotidianas, como gerenciamento financeiro, compras, uso de telefone, administração de medicamentos, entre outras. As respostas variam em uma escala que inclui as opções "Sim, é capaz" (0); "Nunca fez, mas poderia fazer agora" (0); "Com alguma dificuldade, mas faz" (1); "Nunca fez e teria dificuldade agora" (1); "Necessita de ajuda" (2); e "Não é capaz" (3). O escore total, quando igual ou superior a 6 pontos, sugere dependência nas atividades instrumentais de vida diária.
Escala de Katz	Ferramenta utilizada para avaliar a independência funcional em atividades básicas de vida diária (ABVD). Composta por seis itens que abrangem tarefas como banho, vestir-se, ir ao banheiro, transferência, continência e alimentação, a escala classifica o desempenho do indivíduo em cada categoria como independente, com alguma ajuda ou dependente. Os escores variam de 0 a 6, indicando o nível de independência funcional do avaliado, no qual: (0) independente em todas as seis funções; (1) independente em cinco funções e dependente em uma função; (2) independente em quatro funções e dependente em duas funções; (3) independente em três funções e dependente em três funções; (4) independente em duas funções e dependente em quatro funções; (5) independente em uma função e dependente em cinco funções; (6) dependente em todas as seis funções.
Fluência Verbal Semântica (FVS) – animais	Tarefa utilizada para avaliar a função cognitiva, especialmente a linguagem e a memória semântica. O participante é instruído a nomear o maior número possível de animais em um intervalo de tempo específico, geralmente um minuto. O desempenho na tarefa reflete a capacidade do indivíduo de evocar palavras associadas ao tema proposto, sendo uma medida da fluência verbal e da organização semântica. O número total de animais nomeados é contado para determinar o escore na tarefa. As notas de corte consistem em: 9 pontos para analfabetos, 12 pontos para 1 a 8 anos de escolaridade, 13 pontos para 9 anos ou mais de escolaridade.
Teste do Desenho do Relógio (TDR)	Instrumento de avaliação que abrange memória, função motora, função executiva e compreensão verbal. O paciente é instruído a desenhar o visor de um relógio, indicando os ponteiros para 11 horas e 10 minutos. A versão de Shulman et al (1993) estabelece um escore de 0 a 5 pontos da seguinte forma: (0) inabilidade de representar o relógio; (1) desenho relacionado ao relógio, mas com desorganização visuoespacial grave; (2) desorganização visuoespacial moderada com marcação de hora incorreta, perseveração, confusão esquerda-direita, números faltando e/ou repetidos, falta ou excesso de ponteiros; (3) distribuição visuoespacial correta com marcação errada da hora; (4) pequenos erros espaciais com dígitos e hora corretos; (5) relógio perfeito.

## Apêndice 2 Itens de avaliação clínica da deglutição, adaptado de Padovani et al.^(12)^

**Table:**

**Item avaliado**	**Descrição**
Escape oral anterior	Considera-se presente quando há ocorrência de escorrimento do alimento ou líquido pelas comissuras labiais, após a captação do bolo, geralmente por insuficiência do vedamento labial.
Captação alterada do utensílio	Considera-se presente quando ocorrem compensações posturais ou falta de precisão do indivíduo ao levar o utensílio (como copo, colher ou garfo) à boca para realizar a captação do alimento.
Resíduo oral	É o acúmulo de alimento em vestíbulo anterior, lateral, assoalho bucal e/ou superfície lingual após a deglutição. Define-se como presente quando há a observação de resíduos de alimentos na cavidade oral após a deglutição, correspondendo a mais de aproximadamente 25% do volume ofertado.
Tempo de trânsito oral alterado	Considera-se presente quando o intervalo de tempo entre a captação completa do bolo alimentar e o início da elevação do complexo hiolaríngeo, determinado pelo disparo do reflexo de deglutição, excede quatro segundos.
Voz molhada	Considera-se presente quando há a produção de um som borbulhante durante a fonação prolongada do som "e", após a oferta de consistência. Esse fenômeno indica a estase de secreções, líquidos ou alimentos no vestíbulo laríngeo, podendo ser um indicativo de penetração silente nas pregas vocais.
Excursão laríngea reduzida	Considera-se presente quando há capacidade limitada de elevação anterior e superior da laringe (visualmente inferior a dois dedos do examinador) durante a deglutição, o que indica um aumento do risco de aspiração.
Refluxo nasal	Considera-se presente quando ocorre o retorno da consistência para a cavidade nasal durante a deglutição, decorrente de insuficiência no fechamento velofaríngeo
Engasgo	Definido como a obstrução do fluxo aéreo, parcial ou completo, decorrente da entrada de um corpo estranho nas vias aéreas inferiores. Considera-se presente quando há engasgo com difícil recuperação, na ocorrência de tosses durante a deglutição, podendo ocorrer cianose, com difícil recuperação da frequência respiratória de base.
Pigarro	Presente refere-se à sensação ou à ação de aproximação das pregas vocais, manifestando-se como um som de "ahem". Geralmente ocorre devido à presença de muco e/ou alimento na região das pregas vocais.
Tosse	A tosse reflexa durante ou após a deglutição é um sinal de aspiração, sendo indicador da existência de sensibilidade na região laríngea e da habilidade de expectoração. Considera-se presente quando há tosse sem solicitação antes, durante ou após a deglutição.
Ausculta cervical positiva	Considera-se presente quando há ruídos na respiração antes da deglutição que se mantêm com a mesma frequência após a oferta da consistência. Após a deglutição, também é considerada presente se houver a ocorrência de ruídos que não estavam presentes anteriormente.
Queda na SPO2	Considera-se presente quando ocorre uma redução percentual superior a 4% na oxigenação arterial, em relação ao valor basal, após a oferta da consistência. Isso se baseia na hipótese de que a aspiração do alimento pode desencadear um reflexo de broncoespasmo, diminuindo a eficiência da ventilação-perfusão e resultando na diminuição da saturação de oxigênio.
Mastigação alterada	Considera-se presente quando há qualquer alteração na capacidade habitual de triturar os alimentos para transformá-los em partículas menores, facilitando a deglutição e a digestão. Essa alteração pode manifestar-se por meio de movimentos mandibulares descoordenados durante a mastigação e assimetria nos movimentos da mandíbula durante esse processo.

## Apêndice 3 Protocolo de avaliação clínica da deglutição utilizado no serviço

**Table:**

*AVALIAÇÃO CLÍNICA DA DEGLUTIÇÃO: [siglas para preenchimento dos itens: P-presente A-ausente]*			
- ausculta laríngea pré -avaliação: ⬜ negativa ⬜ positiva ________________________________________________________			
- Sinais vitais pré -avaliação FC: ______ bpm (60 a 100 bpm) FR: ______ rpm (12 a 20 rpm) SPO2: ______ % (> 90%)			
**Eventos presentes**	**Néctar**	**Sólido**	**Líquido**
Escape oral anterior			
Resíduo oral			
Captação alterada do utensílio			
Tempo de trânsito oral alterado			
Mastigação alterada			
Excursão laríngea reduzida			
Refluxo nasal			
Engasgo			
Pigarro			
Tosse			
Voz molhada			
Ausculta cervical positiva			
SPO2 (queda acima de 4%)			
Recuperação para saturação de base: ( ) Rápida ( ) Lenta			
Sinais vitais pós-avaliação: FR: _______ FC: ________ SPO2: ___________			
**CONCLUSÃO - FONOAUDIOLOGIA:**			
-Classificação funcional da deglutição -			
Normal ⬜ funcional ⬜ (compensações espontâneas de dificuldades leves em pelo menos uma consistência, com ausência de sinais de risco de aspiração) Disfagia leve ⬜ Disfagia moderada ⬜ Disfagia grave ⬜
