# Supplementary material for: ERRATUM: Characterization of swallowing in older adults with dementia
Source: Codas. 2025 Jun 16;37(3):e20230183. doi: 10.1590/2317-1782/e20230183en (PMC12175593; doi:10.1590/2317-1782/e20230183en)
Supplement: Translation

# Translation: ERRATA: Caracterização da deglutição de idosos com demência

**DOI:** 10.1590/2317-1782/e20230183pt

Devido a erro honesto dos autores, o artigo “Caracterização da deglutição de idosos com demência” (DOI https://doi.org/10.1590/2317-1782/e20230358pt), presente em CoDAS 2025;37(3):e20230358, foi publicado sem a identificação de todos os autores.

**Onde antes se identificava:**

Bruna de Sousa Santos^1^https://orcid.org/0000-0002-6695-9615

Juliana Onofre de Lira^1^https://orcid.org/0000-0001-8938-4781

Laura Davison Mangilli^1^https://orcid.org/0000-0003-2739-126X

Agora, identifica-se:

Bruna de Sousa Santos^1^https://orcid.org/0000-0002-6695-9615

Juliana Onofre de Lira^1^https://orcid.org/0000-0001-8938-4781

Cristina Lemos Barbosa Furia^1^https://orcid.org/0000-0001-9507-6072

Laura Davison Mangilli^1^https://orcid.org/0000-0003-2739-126X

**Onde antes se identificava:**

**Contribuição dos autores**

Concepção e delineamento do estudo: BSS, JOL e LDM; Coleta, análise e interpretação dos dados: BSS, JOL e LDM; Redação ou revisão do artigo de forma intelectualmente importante: BSS, JOL e LDM; Aprovação final da versão a ser publicada: JOL e LDM.

**Agora, identifica-se:**

**Contribuição dos autores**

Concepção e delineamento do estudo: BSS, JOL, CLBF e LDM; Coleta, análise e interpretação dos dados: BSS, JOL, CLBF e LDM; Redação ou revisão do artigo de forma intelectualmente importante: BSS, JOL, CLBF e LDM; Aprovação final da versão a ser publicada: JOL e LDM.

Os autores pedem desculpas pelo erro.
